# Identification and transcriptomic profiling of genes involved in increasing sugar content during salt stress in sweet sorghum leaves

**DOI:** 10.1186/s12864-015-1760-5

**Published:** 2015-07-19

**Authors:** Na Sui, Zhen Yang, Mingli Liu, Baoshan Wang

**Affiliations:** Key Laboratory of Plant Stress Research, College of life science, Shandong Normal University, Jinan, Shandong 250014 PR China

**Keywords:** Transcriptomic profile, Sugar content, Photosynthesis, Salt stress, Sweet sorghum

## Abstract

**Background:**

Sweet sorghum is an annual C4 crop considered to be one of the most promising bio-energy crops due to its high sugar content in stem, yet it is poorly understood how this plant increases its sugar content in response to salt stress. In response to high NaCl, many of its major processes, such as photosynthesis, protein synthesis, energy and lipid metabolism, are inhibited. Interestingly, sugar content in sweet sorghum stems remains constant or even increases in several salt-tolerant species.

**Results:**

In this study, the transcript profiles of two sweet sorghum inbred lines (salt-tolerant M-81E and salt-sensitive Roma) were analyzed in the presence of 0 mM or 150 mM NaCl in order to elucidate the molecular mechanisms that lead to higher sugar content during salt stress. We identified 864 and 930 differentially expressed genes between control plants and those subjected to salt stress in both M-81E and Roma strains. We determined that the majority of these genes are involved in photosynthesis, carbon fixation, and starch and sucrose metabolism. Genes important for maintaining photosystem structure and for regulating electron transport were less affected by salt stress in the M-81E line compared to the salt-sensitive Roma line. In addition, expression of genes encoding NADP^+^-malate enzyme and sucrose synthetase was up-regulated and expression of genes encoding invertase was down-regulated under salt stress in M-81E. In contrast, the expression of these genes showed the opposite trend in Roma under salt stress.

**Conclusions:**

The results we obtained revealed that the salt-tolerant genotype M-81E leads to increased sugar content under salt stress by protecting important structures of photosystems, by enhancing the accumulation of photosynthetic products, by increasing the production of sucrose synthetase and by inhibiting sucrose decomposition.

**Electronic supplementary material:**

The online version of this article (doi:10.1186/s12864-015-1760-5) contains supplementary material, which is available to authorized users.

## Background

Soil salinity is not only one of the major factors leading to deterioration of the ecological environment but also a major abiotic stress in plant agriculture worldwide [1]. Salt stress involves a combination of osmotic stress and ionic stress that greatly affects plant growth and crop production [2]. Upon salt treatment, lots of the major processes within plants, such as photosynthesis, protein synthesis, energy metabolism and lipid metabolism are affected [3]. Salt treatment also regulates the expression level of many genes involved either directly or indirectly in plant protection [2]. In the past few decades, many efforts have been made to understand the molecular mechanisms of salt tolerance. Utilization of genes related to compatible solutes [4], ion transporters [5] and transcription factors [6] is regarded as a way to improve the salt tolerance of plants.

Sweet sorghum [*Sorghum bicolor* (L.) Moench] which originates from Africa is an annual C4 crop [7]. Sweet sorghum has a fast growth rate and high efficiency of biomass accumulation. It is consumed as a food source for humans and as livestock feed. In addition, it has been considered to be one of the most promising bio-energy crops [8], as the stalks are rich in fermentable sugars. The tolerance of sweet sorghum to salinity is thought to be high. However, there are salt-tolerant and salt-sensitive genotypes of sweet sorghum. Salt-tolerant genotypes have a greater ability to exclude toxic ions and to store the absorbed toxic ions in the root cell vacuoles while maintaining high levels of K^+^ uptake. As a result, the accumulation of Na^+^ and Cl^−^ in actively growing shoots and leaves may be limited. This mechanism can effectively prevent the photosynthetic apparatus of sweet sorghum from being damaged by Na^+^ and Cl^−^. On the other hand, the ability to compartmentalize Na^+^ within root cell vacuoles is lower in salt-sensitive genotypes [9], which results in a higher level of Na^+^ accumulated in leaves. Due to this accumulation, the photosynthetic apparatus may be damaged by Na^+^ and the photosynthesis will significantly decrease.

Interestingly, it has been shown that the brix of salt-sensitive sweet sorghum decreases under salt stress. The brix of salt-tolerant species, on the other hand, stays stable or is even increased by salt stress [10–12]. As we know, the main source of carbon and energy in the sink tissues of sweet sorghum is sucrose. Several physiological processes play important roles in maintaining the high sugar content in stems of sweet sorghum. **A**) CO_2_ from the atmosphere is fixed in the mesophyll cells. Stalk sugar content accumulation of sweet sorghum depends on the synthesis and accumulation of photosynthetic products. The initial product of CO_2_ fixation is oxaloacetate (OAA). OAA is converted into a transportable form (malate) and is then transported to the bundle sheath. After a series reactions through the C4 pathway, 3-phosphoglycerate is produced which is then converted to triose phosphate (TP) [13]. **B**) Once TP has been produced, it either leaves the chloroplast via the triose phosphate translocator (TPT) in exchange for *ortho*-phosphate or it remains in the chloroplast stroma for the completion of the Calvin cycle or to be converted to starch [14]. **C**) TP in the cytoplasm can be converted to fructose-6-phosphate (Fru-6-P). Then, Fru-6-P can be further converted to sucrose by sucrose phosphate phosphatase (SPS, EC3.1.2.24) or to UDP-glucose (UDP-Glu). UDP-Glu is used as substrates in sucrose synthesis, a reaction catalyzed by sucrose synthase (SS, EC2.4.1.13). Sucrose can be decomposed into glucose and fructose by invertase (INV, EC3.2.1.26) in vacuole. **D**) Six sucrose transporters (SUT1–6) have been reported in monocots [15], which are located in the plasma membranes of sieve elements and companion cells, or in tonoplasts of storage cells. SUTs have been reported to play an important role in the re-distribution of sucrose [16].

Although there are numerous studies on the response mechanism of sweet sorghum to salt stress, most of which are restricted to the eco-physiological level or to the study of a single pathway. The physiological and molecular mechanisms of increasing sugar content in salt-tolerant sweet sorghum species under salt stress is remain unclear. In recent years, with the increasing availability of sequence data, expression profiling has been used to identify genes involved in the adaptive responses abiotic stresses. A common strategy to identify genes related to salt stress is using a comparative study of different genotypes or cultivars in the tolerance to the abiotic stress [17–19]. Comparisons between salt-sensitive and salt-tolerant genotypes of model and non-model plant species have been reported, including Arabidopsis [19], rice [18, 20], olive [17], populus [21] and tomato [22]. In the present study, the transcriptomes of salt-sensitive and a salt-tolerant sweet sorghum inbred lines were analyzed by high-throughput Illumina RNA-sequencing (RNA-seq). By comparing the transcriptomes of a salt-sensitive and a salt-tolerant sweet sorghum inbred line under salt stress, we identified 864 and 930 differentially expressed genes (DEGs) between control plants and those subjected to salt in M-81E and Roma, respectively. Results of this study should provide further insight into the complex regulatory networks underlying the mechanism of higher sugar content under salt stress in sweet sorghum.

## Results

### Effects of salt stress on growth parameters

**Fig. 1:**
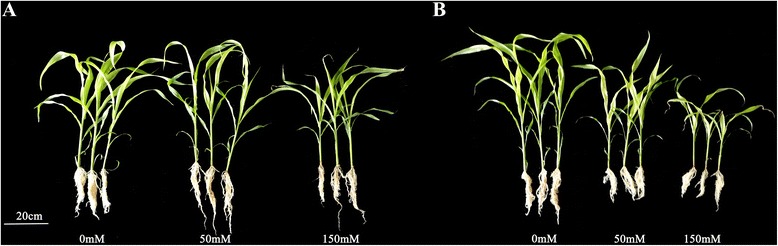
The phenotype of M-81E (**a**) and Roma (**b**) treated with different concentrations of NaCl (0, 50 and 150 mM) for 7 days

After treated with 50 mM NaCl for 7 days, there was no significant difference in M-81E (Fig. 1a), while growth of Roma was significantly inhibited (Fig. 1b). In the presence of 150 mM NaCl, the growth of both genotypes was inhibited, but it was more severe in Roma. Leaf length of M-81E was not affected by 50 mM NaCl treatment, but slightly decreased 15.6 % at 150 mM NaCl treatment. Leaf length of Roma decreased 27.2 % at 50 mM NaCl treatment and 41.6 % at 150 mM NaCl treatment (Additional file 1: Figure S1). Leaf numbers of M-81E and Roma were not affected by 50 mM NaCl, but decreased 23.2 and 31.3 %, respectively, when treated with 150 mM NaCl (Additional file1: Figure S1). Fresh weight (FW) of leaves of both genotypes gradually decreased with an increase in NaCl concentration. The reductions were more severe at 150 mM, particularly for Roma (Additional file 2: Figure S2) in which values decreased 43.1 and 68.6 % for 50 and 150 mM NaCl concentrations, respectively. Dry weight (DW) of leaves also decreased with an increase in NaCl concentration. The highest reduction in Roma was 62.9 % at 150 mM NaCl (Additional file 2: Figure S2). There was no significant effect on water content during NaCl treatment. (Additional file 2: Figure S2).

### Effects of salt stress on ion concentration

After treated with 50 mM NaCl for 7 days, there were no significant changes in Na^+^ concentrations in leaves of both genotypes compared to control plants (Additional file 3: Figure S3). When a higher concentration of salt (150 mM) was applied, Na^+^ concentration increased significantly, especially for Roma. The K^+^ concentration in leaves gradually decreased in response to NaCl. At the 150 mM NaCl treatment, K^+^ concentration of M-81E and Roma decreased 30.6 and 41.6 %, respectively (Additional file 3: Figure S3). The K^+^/Na^+^ ratio in leaves of M-81E increased under 50 mM NaCl treatment and then decreased when treated by 150 mM NaCl. While the K^+^/Na^+^ ratio in leaves of Roma decreased under the NaCl treatment. At 150 mM NaCl treatment, the K^+^/Na^+^ ratio in Roma decreased by a factor of fourteen times (Additional file 3: Figure S3).

### Effects of salt stress on PSII photochemical efficiency

**Fig. 2:**
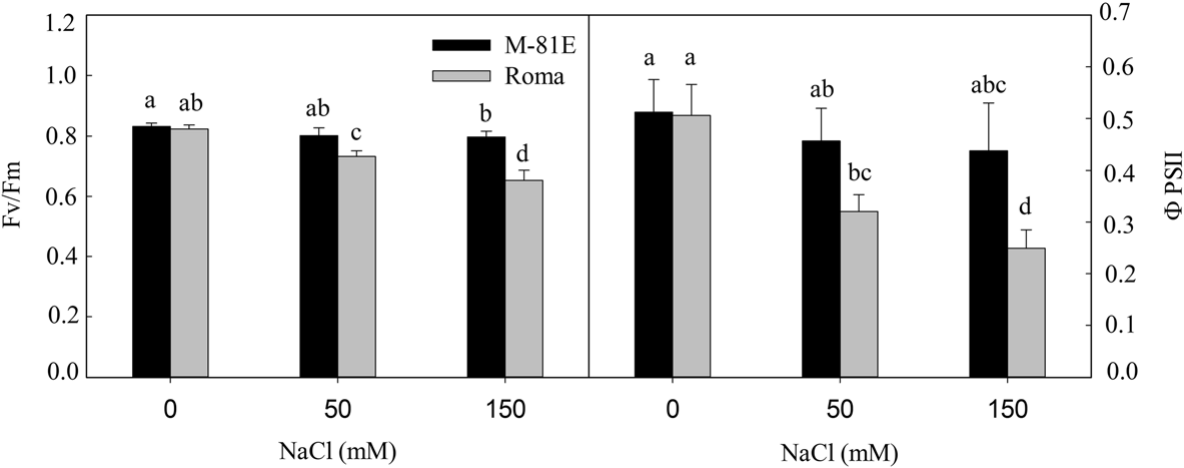
Effect of salt stress (0, 50 and 150 mM) on Fv/Fm and ΦPSII in leaves of M-81E and Roma. Fv/Fm and ΦPSII were measured after treated with NaCl for 7 days. Values are means ± SD of five measurements for each of five plants. Bars with the different letters are significantly different at *p* = 0.05 according to Duncan’s multiple range test. Bars with same letter are not significantly different

In both genotypes the potential efficiency of PSII photochemistry (Fv/Fm) was reduced with increasing NaCl concentration (Fig. 2). After treated with 50 mM NaCl for 7 days, Fv/Fm of M-81E and Roma decreased 3.6 and 11.1 %, respectively. For 150 mM NaCl, Fv/Fm of M-81E and Roma decreased 4.2 and 20.8 %, respectively (Fig. 2). The actual PSII efficiency (ΦPSII) decreased in both genotypes after treated with NaCl. ΦPSII of M-81E treated with 50 and 150 mM NaCl decreased 10.7 and 14.4 %, respectively. In Roma, ΦPSII decreased 36.6 and 50.7 % for 50 and 150 mM NaCl treatment, respectively.

### Effects of salt stress on chlorophyll content

**Fig. 3:**
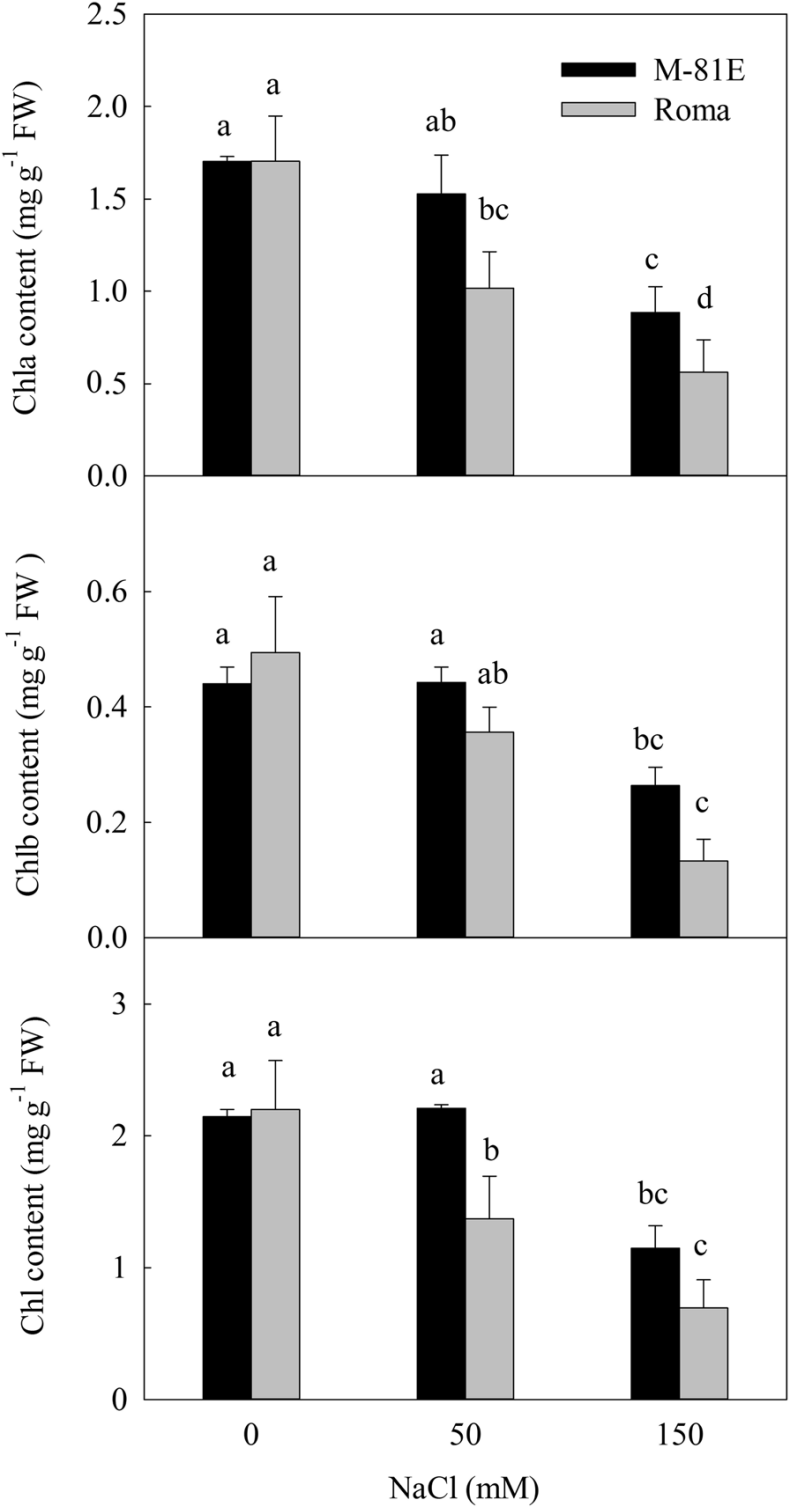
Chlorophyll content of M-81E and Roma treated with different concentrations of NaCl (0, 50 and 150 mM) for 7 days. Values are means ± SD of five replicates. Bars with the different letters are significantly different at *p* = 0.05 according to Duncan’s multiple range test. Bars with same letter are not significantly different

The effects of increasing level of NaCl salinity on chlorophyll contents in the two genotypes were determined after 7 day exposure to salinity (Fig. 3). Chlorophyll content in M-81E was not changed significantly by 50 mM NaCl but decreased 46.5 % under 150 mM NaCl treatment. On the other hand, in Roma, chlorophyll content decreased gradually with the increasing NaCl treatments. Chlorophyll content of Roma treated with 50 and 150 mM NaCl decreased 37.6 and 68.4 %, respectively.

### Effects of salt stress on photosynthesis

**Fig. 4:**
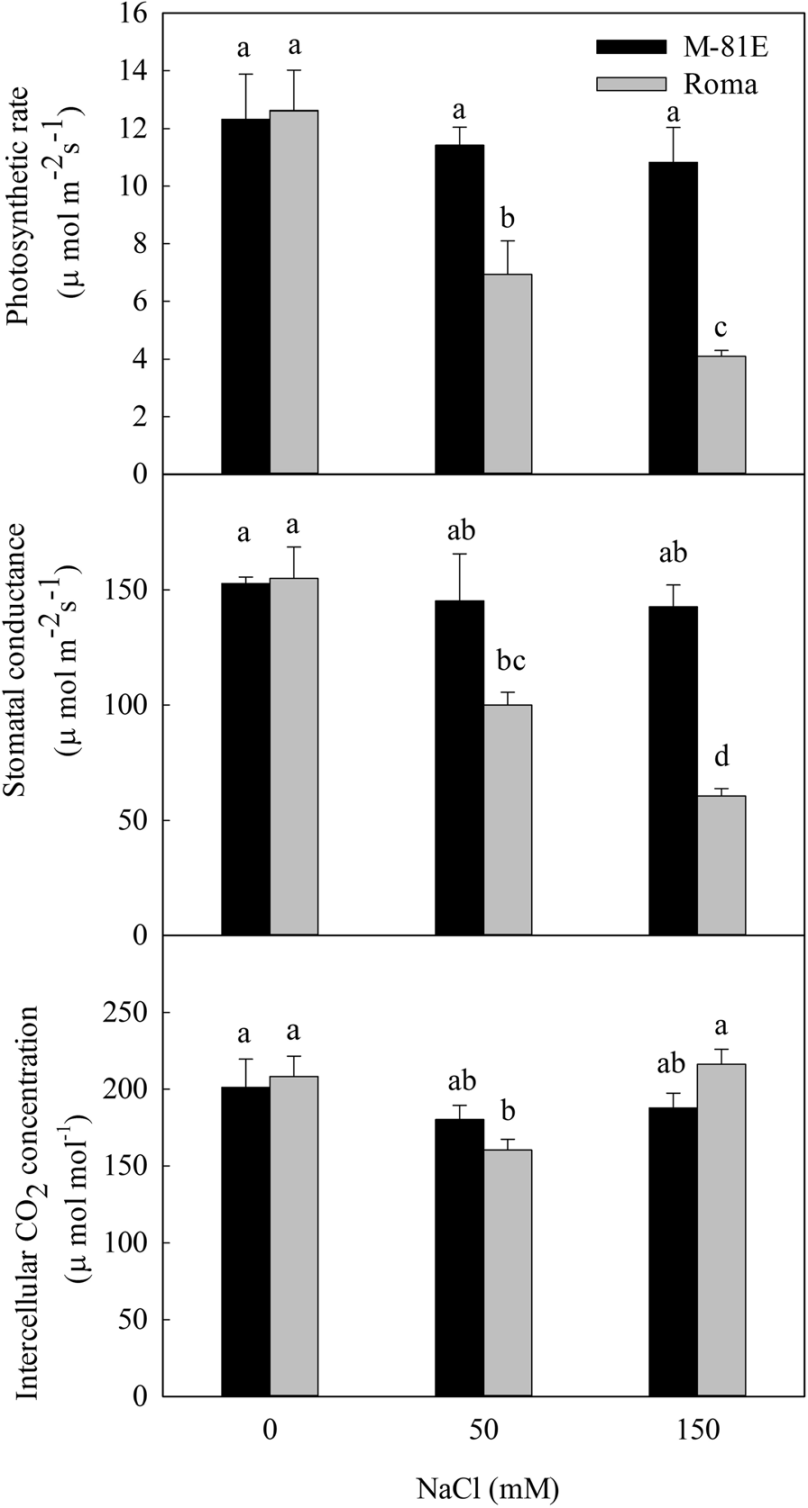
Photosynthetic rate, stomatal conductance and intercellular CO_2_ concentration of M-81E and Roma treated with different concentrations of NaCl (0, 50 and 150 mM) for 7 days. Values are means ± SD of five replicates. Bars with the different letters are significantly different at *p* = 0.05 according to Duncan’s multiple range test. Bars with same letter are not significantly different

There were no significant changes in photosynthetic rate, stomatal conductance and intercellular CO_2_ concentration in M-81E under salt stress. However, photosynthesis in Roma was significantly influenced by salt stress (Fig. 4). The photosynthetic rate of Roma was inhibited after treated with NaCl for 7 days. The reduction percentage of photosynthetic rate of Roma was 45.1 and 67.5 % for 50 mM and 150 mM NaCl treatment, respectively. Stomatal conductance of Roma decreased 35.5 and 60.9 % after treated with 50 mM and 150 mM NaCl, respectively. Intercellular CO_2_ concentration of Roma decreased 23.0 % under 50 mM NaCl. While after treated with 150 mM NaCl for 7 days, the intercellular CO_2_ concentration of Roma increased 3.9 %.

### Effects of salt stress on sugar content

**Fig. 5:**
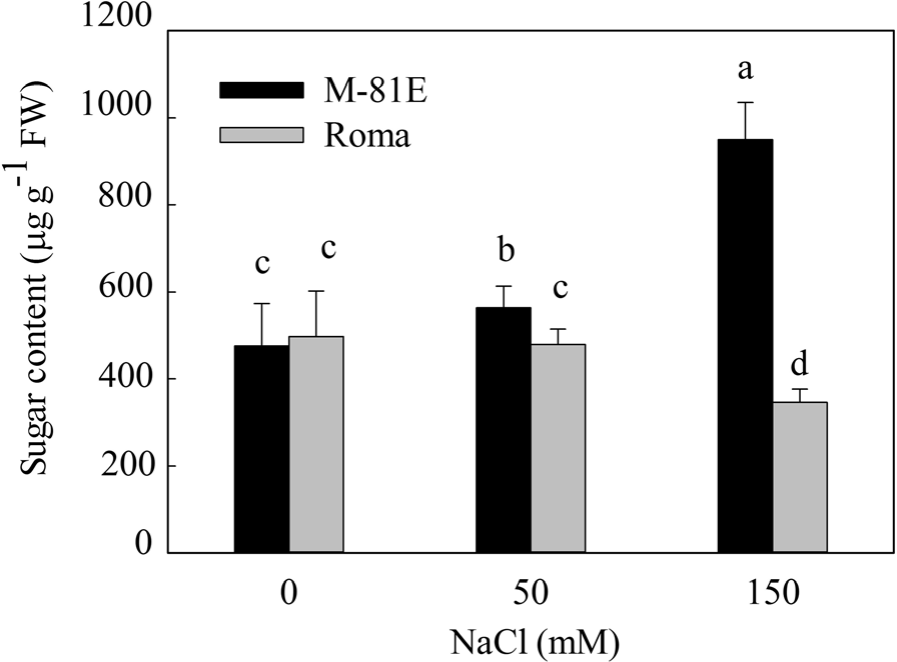
Sugar content of M-81E and Roma treated with different concentrations of NaCl (0, 50 and 150 mM) for 7 days. Values are means ± SD of five replicates. Bars with the different letters are significantly different at *p* = 0.05 according to Duncan’s multiple range test

The effects of increasing level of NaCl salinity on sugar contents in the two genotypes were determined after 7 days exposure to salinity. After treated for 7 days, the sugar content of M-81E increased 15.6 and 99.7 % under 50 mM and 150 mM NaCl, respectively. While, there was no significant change in sugar content of Roma under 50 mM NaCl. Under 150 mM NaCl, the sugar content of Roma decreased 30.5 % (Fig. 5).

### Sequencing output and assembly

In order to investigate the molecular mechanisms of high sugar content under salt stress in sweet sorghum, libraries (MC, MS, RC and RS) were designed for RNA-seq. MC and MS libraries were used for leaves of M-81E treated with 0 mM and 150 mM NaCl, respectively. RC and RS libraries were used for leaves of Roma treated with 0 mM and 150 mM NaCl, respectively. In total, 78.41 million reads were generated. After trimming adapters and filtering out low quality reads, more than 67.08 million clean reads were retained for assembly and further analysis. Among all the reads, more than 94 % had Phred-like quality scores at the Q30 level (an error probability of 0.1 %) (Additional file 4: Table S1). All these data showed that the throughput and sequencing quality were high enough for further analysis. The reads produced in this study have been deposited in the National Center for Biotechnology Information (NCBI) SRA database and accession number was shown in “Availability of supporting data”.

### Exploration of DEGs in response to salt stress

**Fig. 6:**
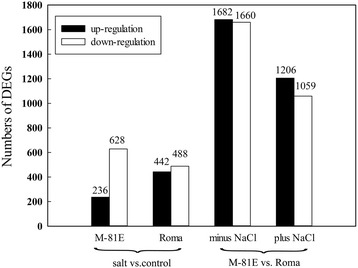
Numbers of DEGs of different genotypes affected by salt stress

In the absence of salt, 3342 genes showed differential expression levels when comparing M-81E vs. Roma. While in the presence of salt, the DEGs between them were 2265. For M-81E, 864 genes were differentially expressed between control plants and those subjected to salt. Among these DEGs, 236 genes were up-regulated in leaves under salt stress. For Roma, 930 genes were differentially expressed between control plants and those subjected to salt. Among these DEGs, 442 genes were up-regulated in leaves under salt stress (Fig. 6). All of these DEGs were selected for further analysis.

### Functional categorization of stress-regulated genes

#### Functional classification by GO

**Fig. 7:**
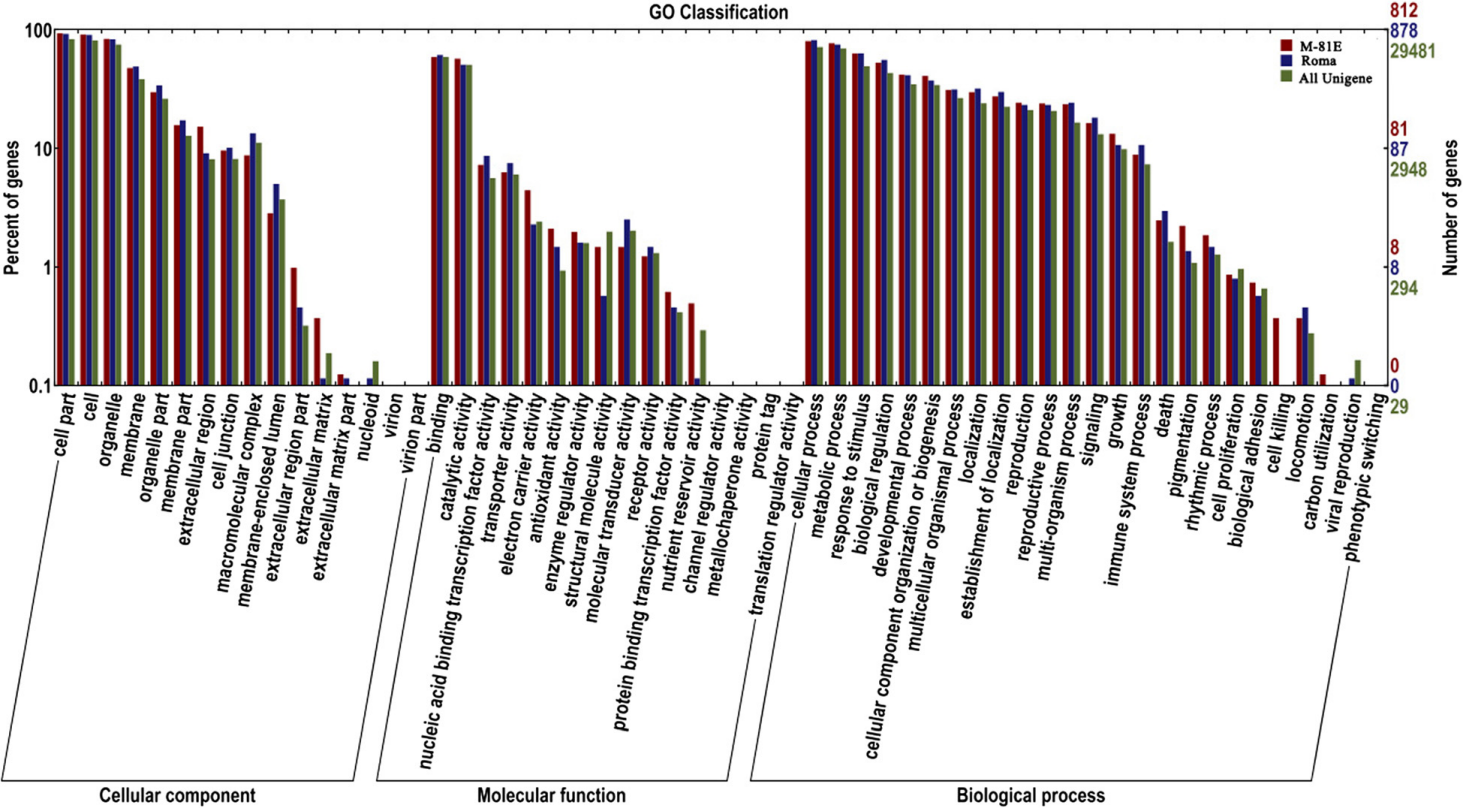
Functional annotation of assembled sequences based on gene ontology (GO) categorization. Results are summarized for three main Go categories: Biological Process, Molecular Function, and Cellular Component

In order to assign functional information to the DEGs between control plants and those treated with NaCl, Gene Ontology (GO) [23] analysis was carried out. This analysis provides a dynamic, controlled vocabulary and also hierarchical relationships for the representation of information on biological processes, molecular function, and cellular components, forming a coherent annotation of various gene products [23]. In M-81E, there were 812 unique transcripts assigned to 48 level-2 GO terms, which were summarized under three main GO categories, including 13 for cellular component, 12 for molecular function and 23 for biological process, respectively. In Roma, there were 878 unique transcripts assigned to 47 level-2 GO terms including 13 for cellular component, 12 for molecular function and 22 for biological process, respectively. For the cellular group, in both M-81E and Roma, the most represented category was cell part, cell and organelle. For molecular function, the category of binding was the most represented GO term, followed second by the category of catalytic activity. Regarding biological process, NCBI UniGene for cellular process and metabolic process were highly represented (Fig. 7).

#### Functional classification by COG

In addition, all the DEGs were subjected to a search against the Clusters of Orthologous Groups (COG) [24] classification. Among the 864 DEGs, 349 sequences showed a COG classification in M-81E (Additional file 5: Figure S4A). Among the 25 COG categories, the cluster for “general function prediction only” was the largest group, followed by “secondary metabolites biosynthesis, transport and catabolism”, “amino acid transport and metabolism”, “carbohydrate transport and metabolism” and “transcription”. The categories “chromatin structure and dynamics”, “extracellular structure” and “nuclear structure” had no corresponding genes. The 360 sequences of the 930 sequences could be assigned to COG classifications in Roma (Additional file 5: Figure S4B). The cluster for “general function prediction only” represented the largest group, followed by “signal transduction mechanisms”, “transcription”, “replication, recombination and repair” and “carbohydrate transport and metabolism”. Whereas no unigenes were assigned to “extracellular structure”, “nuclear structure”, “cell motility” and “intracellular trafficking, secretion, and vesicular transport”.

#### Functional classification by KEGG

**Fig. 8:**
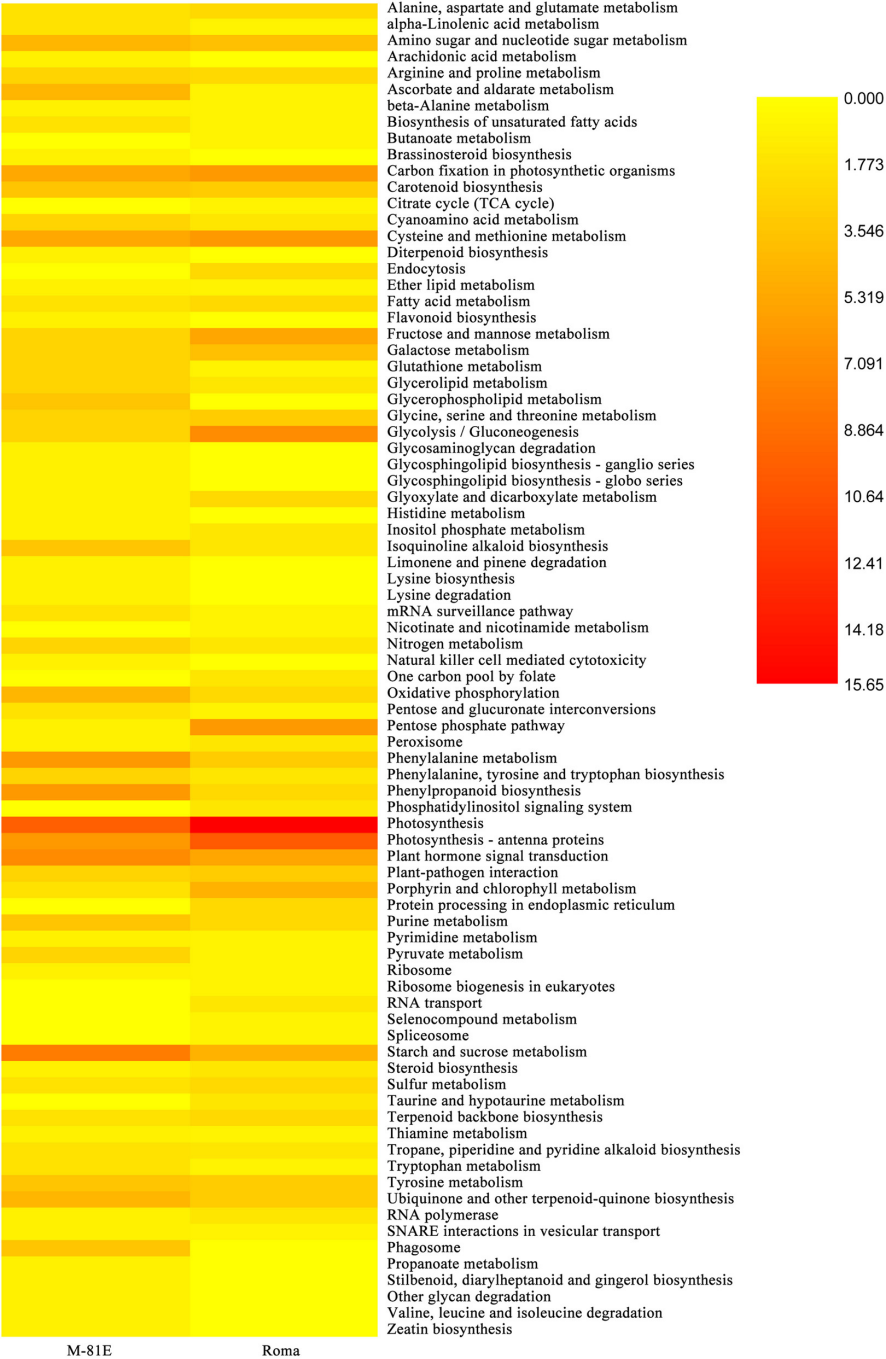
The heat map display of DEGs assigned to different KEGG pathways. The numbers in the scale bar show the percentage of the number of DEGs assigned to a certain KEGG pathway in which assigned to all KEGG pathways. Red indicates that more genes are enriched in this pathway

Kyoto Encyclopedia of Genes and Genomes database (KEGG) [25] was used to identify potential biological pathways represented in the sweet sorghum transcriptome. There were 150 DEGs of M-81E and 174 DEGs of Roma assigned to 70 and 63 KEGG pathways, respectively. The majority of these DEGs mapped to “photosynthesis”, “photosynthesis-antenna proteins”, “carbon fixation in photosynthetic organisms” and “starch and sucrose metabolism” categories (Fig. 8, Table 1), which indicated that salt stress mainly affected photosynthesis and carbohydrate metabolism in leaves of sweet sorghum.

**Table 1:** DEGs mapped to KEGG pathways related with sugar content

Gene ID	Annotation	M-81E			Roma		
		FDR	Log2FC	regulated	FDR	Log2FC	regulated
A: Photosynthesis - antenna proteins							
Sb01g015400	Chlorophyll a-b binding protein	1.63E-05	−1.61	down	2.66E-07	−2.15	down
Sb02g032040	Chlorophyll a-b binding protein 1	-	-	-	5.56E-16	−2.47	down
Sb02g036260	Chlorophyll a-b binding protein CP29.1	1.56E-14	−1.5	down	0	−1.38	down
Sb02g036380	Chlorophyll a-b binding protein	4.11E-14	−2.37	down	1.39E-06	−1.62	down
Sb02g037410	Chlorophyll a-b binding protein 7	-	-	-	0	−1.36	down
Sb03g027030	Chlorophyll a-b binding protein 2	-	-	-	1.44E-24	−3.45	down
Sb03g027040	Chlorophyll a-b binding protein 2	6.22E-35	−2.41	down	4.37E-17	−2.78	down
Sb04g004770	Chlorophyll a-b binding protein 1B-21	2.35E-12	−1.48	down	0	−1.54	down
Sb05g007070	Chlorophyll a-b binding protein CP26	5.84E-16	−2.01	down	1.95E-05	−1.68	down
Sb06g032690	Chlorophyll a-b binding protein CP24 10B	-	-	-	6.29E-09	−1.62	down
Sb07g021260	Chlorophyll a-b binding protein 4	-	-	-	2.55E-05	−1.74	down
Sb09g028720	Chlorophyll a-b binding protein M9	3.80E-05	−1.42	down	0	−1.36	down
Sb10g023930	Chlorophyll a-b binding protein 8	-	-	-	0	−1.49	down
Sb01g015400	Chlorophyll a-b binding protein	1.63E-05	−1.61	down	2.66E-07	−2.15	down
B: Photosynthesis							
Sb01g004330	Photosystem I reaction center subunit II	-	-	-	1.05E-05	−1.62	down
Sb01g006370	Photosystem I reaction center subunit III	-	-	-	0.01	−1.1	down
Sb01g012850	Ferredoxin	-	-	-	0	−1.11	down
Sb01g036240	Photosystem II repair protein PSB27-H1	7.37E-12	−1.37	down	1.05E-05	−1.35	down
Sb02g002830	Photosystem II 10 kDa polypeptide, chloroplastic (Precursor)	5.40E-09	2.16	up	-	-	-
Sb02g002960	Photosystem I reaction center subunit psaK	6.29E-14	−1.5	down	1.25E-06	−1.83	down
Sb02g010190	Photosystem I reaction center subunit IV	-	-	-	0	−1.22	down
Sb02g035610	Oxygen-evolving enhancer protein 3-1	9.12E-09	−1.15	down	0	−1.12	down
Sb02g027900	Photosystem I reaction center subunit V	-	-	-	0	−1.7	down
Sb02g034570	ATP synthase subunit gamma	-	-	-	0.01	−1	down
Sb03g004560	Photosystem I reaction center subunit XI	-	-	-	0	−1.41	down
Sb03g036090	Photosystem II reaction center W protein	-	-	-	0	−1.39	down
Sb04g023940	PsbQ-like protein 1	-	-	-	0.01	−1.07	down
Sb04g027810	ATP synthase delta chain	1.22E-08	−1.15	down	0	−1.07	down
Sb06g016090	hypothetical protein SORBIDRAFT_06g016090	2.50E-07	−1.05	down	1.88E-09	−2.04	down
Sb07g000600	Ferredoxin-1	1.83E-15	−1.55	down	0	−2.02	down
Sb07g000610	Ferredoxin-1	-	-	-	3.78E-05	−1.2	down
Sb07g000620	Ferredoxin-1	0	−1.54	down	0.01	−1.22	down
Sb09g021810	Ferredoxin-6	0	1.69	up	-	-	-
Sb08g005300	Photosystem I reaction center subunit N	-	-	-	1.74E-05	−1.49	down
Sb09g028260	Photosystem I reaction center subunit VI	6.72E-07	−1.02	down	6.56E-06	−1.66	down
Sb10g000230	Plastocyanin	5.08E-09	−1.25	down	0	−1.32	down
C:Carbon fixation in photosynthetic organisms							
Sb02g004280	Probable ribose-5-phosphate isomerase	-	-	-	5.79E-05	−1.2	down
Sb03g043140	Fructose-bisphosphate aldolase	-	-	-	5.10E-05	1.27	up
Sb05g003480	Ribulose bisphosphate carboxylase small chain	2.33E-09	−1.49	down	0	−1.36	down
Sb05g004590	Fructose-bisphosphate aldolase	-	-	-	6.28E-08	−1.73	down
Sb06g004280	Transketolase	1.02E-12	Inf	up	0	2.64	up
Sb10g002220	Transketolase	-	-	-	0	−1.05	down
Sb10g026710	Phosphoglycerate kinase	-	-	-	0	1.98	up
Sb01g023750	Alanine aminotransferase 2	1.00E-11	1.37	up	-	-	-
Sb03g034280	NADP-dependent malic enzyme	4.64E-05	Inf	up	-	-	-
Sb06g018880	Glyceraldehyde-3-phosphate dehydrogenase A,	0	−1.08	down	-	-	-
D:starch and sucrose metabolism							
Sb01g035890	Sucrose synthase 4	5.12E-05	1.92	up	-	-	-
Sb02g020410	Glucose-1-phosphate adenylyltransferase small subunit	2.04E-05	2.02	up	-	-	-
Sb03g012830	Pectinesterase 1 (Precursor)	3.45E-09	-Inf	down	-	-	-
Sb04g021540	1,4-alpha-glucan-branching enzyme 2	0	1.32	up	-	-	-
Sb06g022410	Beta-glucosidase 16 (Precursor)	1.84E-08	−1.65	down	-	-	-
Sb06g022450	Probable inactive beta-glucosidase 14 (Precursor)	0.01	−2.73	down	-	-	-
Sb06g023760	Beta-fructofuranosidase 1 (Precursor)	9.66E-06	−1.56	down	5.28E-06	1.44	up
Sb09g005840	Hexokinase-7	1.69E-07	−1.47	down	0	1.49	up
Sb09g025790	Alpha,alpha-trehalose-phosphate synthase [UDP-forming]	1.03E-06	−1.36	down	-	-	-
Sb01g007580	UDP-glucose 6-dehydrogenase 5	-	-	-	0	1.23	up
Sb08g019260	Probable galacturonosyltransferase 13	-	-	-	0	1.07	up
Sb09g022050	Probable beta-D-xylosidase 2 (Precursor)	-	-	-	4.87E-11	2.13	up
Sb09g029610	Glucose-1-phosphate adenylyltransferase large subunit	-	-	-	1.16E-07	1.58	up

DEGs mapped to photosynthesis-antenna proteins, photosynthesis, carbon fixation in photosynthetic organisms and starch and sucrose metabolism pathway. “Inf” means Infinite, “-” means the expression of the gene was not changed under salt stress

### Photosynthesis-antenna proteins

In the first steps of photosynthesis, light energy is captured and converted into chemical energy. A large part of the light is absorbed by the outer light-harvesting complexes (LHCs), which contain most of the chlorophyll and carotenoid pigments and are peripherally associated with PSI and PSII [26, 27]. These LHC proteins are encoded by nuclear genes of the LHC multi-gene family coding for proteins that contain one to four trans-membrane helices and share a number of conserved chlorophyll- and xanthophyll-binding motifs [28]. In higher plants, 14 different types of LHC proteins (Lhca1–Lhca6 and Lhcb1–Lhcb8) are expressed [29]. Lhca-type proteins are organized into two heterodimeric domains (Lhca1/Lhca4 and Lhca2/Lhca3) as an external antenna with the PSI core. The reaction center of PSII is surrounded by Lhcb-type proteins. In the present study, 8 DEGs of M-81E and 14 DEGs of Roma were mapped to the antenna proteins, respectively. In comparison with the untreated control, the expression of DEGs encoding Lhca1 and Lhcb1-5 were down-regulated in both of the two genotypes under salt stress. However, the expression level of DEGs encoding Lcha2-4 and Lchb6 dropped under salt stress in Roma but did not change in M-81E (Additional file 6: Figure S5).

### Photosynthesis

**Fig. 9:**
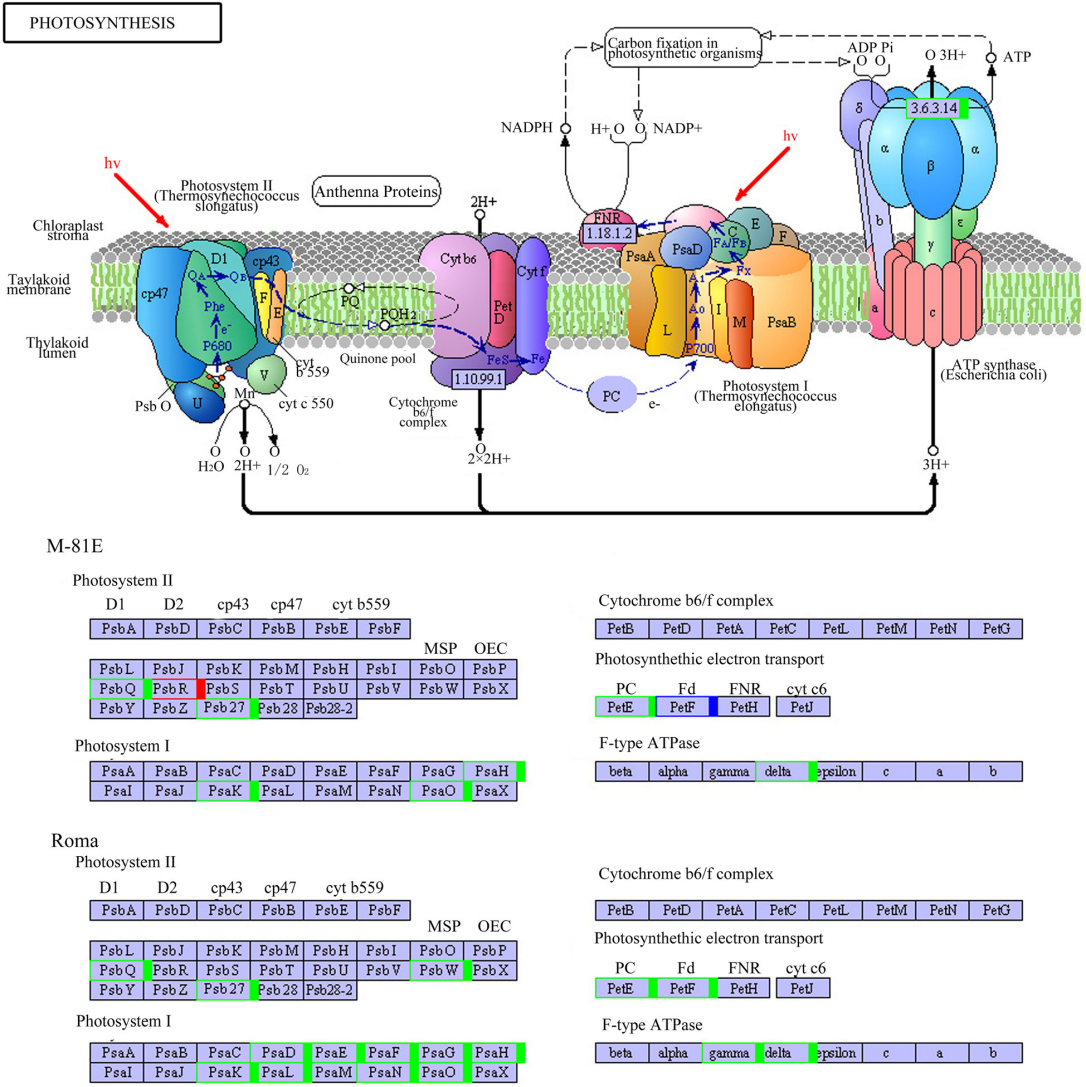
KEGG map of the photosynthesis pathway. It’s an analysis of DEGs, comparing salt-treated samples to untreated control. Boxes with a red frame indicate the corresponding DEGs were up-regulated in the salt-treated samples, boxes with a green frame indicate the corresponding DEGs were down-regulated in the salt-treated samples, boxes with blue frame indicate some of the corresponding DEGs were down-regulated and others were up-regulated, and those without any colored frame indicate the expression level of corresponding genes were not changed, as determined by RNA-seq

Photosynthesis is one of the most important metabolic processes in plants. Salt stress significantly impacts the photosynthetic rate [30, 31]. The four protein components of the photosynthetic electron transport chain responsible for the electron transfer from water to NADP^+^ are Photosystem II (PSII), Photosystem I (PSI), cytochrome (Cytb6f) complex, and ATP synthase. There were 11 and 20 DEGs of M-81E and Roma, respectively, that mapped to the photosynthesis pathway, which led to changes in the structure and function of the four protein components (Fig. 9, Table 1).

Photosystem II is a protein complex consisting of several different types of chlorophyll binding components. The function of these components is to organize chlorophylls for light harvesting and to harbor the electron transport intermediates as well as cofactors needed for the oxidation of water [32]. After treated with NaCl, DEGs encoding PsbQ, which is necessary for regulation of activity and assembly [33, 34] of PSII in both M-81E and Roma, were down-regulated. PsbR has been proved to be an important link in the PSII core complex to permit stable assembly of the oxygen-evolving complex proteins PsbP and PsbQ [35]. DEGs encoding PsbR were up-regulated in M-81E after treated with 150 mM NaCl for 48 h. DEGs encoding PsbW, which stabilize the supramolecular organization of photosystem II, were down-regulated only in Roma. These results suggested that salt stress reduced the binding stability of several subunits of PSII. However, we predict that M-81E may protect important connective structures from being destroyed by increasing expression of specific genes.

Photosystem I (PSI) from higher plants is a supramolecular complex which catalyzes the light-driven electron transfer from plastocyanin to ferredoxin and is composed of a chlorophyll binding core complex and a chlorophyll a/b binding peripheral antenna called LHCI [36]. After treated with NaCl for 48 h, the expression of DEGs encoding PsaK, PsaH and PsaO decreased in both genotypes. All of these three subunits are involved in the interaction between the light-harvesting complex (LHC) and Photosystem I [37–39], suggesting that salt stress weakened the connection between LHCs and PSI and reduced the conversion of light energy to chemical energy. PsaD, PsaE, PsaF, PsaG, PsaL and PsaN encoding genes were down-regulated only in Roma. Among them, four subunits (PsaD, PsaE, PsaF, PsaN) are considered to be important for the interaction with ferredoxin or plastocyanin [40, 41], indicating that the electron transport mechanism was inhibited by salt stress in Roma. These observations agreed fairly well with the down-regulation of *petE* and *petF* in Roma after treated with salt. Moreover, expression of *Sb04g027810*, a gene encoding the ATP synthase delta chain, decreased in both genotypes when treated with 150 mM NaCl, while the gene encoding the ATP synthase gamma chain was only down-regulated in Roma.

### Carbon fixation in photosynthetic organisms

There were 6 and 8 DEGs of M-81E and Roma, respectively, mapped to the carbon fixation in photosynthetic organisms pathway. Ribulose-bisphosphate carboxylase (rubisco, EC: 4.1.1.39), phosphoenolpyruvate carboxylase (PEPC, EC:4.1.1.31) and pyruvate orthophosphate dikinase (PPDK, EC:2.7.9.1) are considered as key enzymes in the process of carbon fixation. Rubisco catalyzes the incorporation of CO_2_ into ribulose 1,5-bisphosphate [42]. Under salt stress for 48 h, the expression of DEGs encoding rubisco decreased while the PPDK and PEPC encoding genes remained unchanged in both genotypes based on our RNA-seq data. Surprisingly, the expression of DEGs encoding transketolase (EC:2.2.1.1) and NADP^+^-malate dehydrogenase (NADP-ME, 1.1.1.40) in M-81E were extremely enhanced by salt stress (Additional file 7: Figure S6).

### Starch and sucrose metabolism

Sucrose phosphate synthetase (SPS, EC:3.1.3.24), sucrose synthetase (SS, EC:2.4.1.13) and invertase (INV, EC:3.2.1.26) are considered to be key enzymes in sucrose metabolism. SS is known to play a role in sucrose synthesis using uridine diphosphate (UDP)-glucose and fructose as substrates and its activity is high in source tissues such as leaves [43]. After a 48 h treatment with NaCl, the expression of DEGs encoding SS were enhanced in M-81E but unchanged in Roma. INV plays the most important role in the decomposition of sucrose. In the present study, the expression of DEGs encoding INV decreased in M-81E but increased in Roma during salt stress (Additional file 8: Figure S7).

### Verification of RNA-seq data

**Fig. 10:**
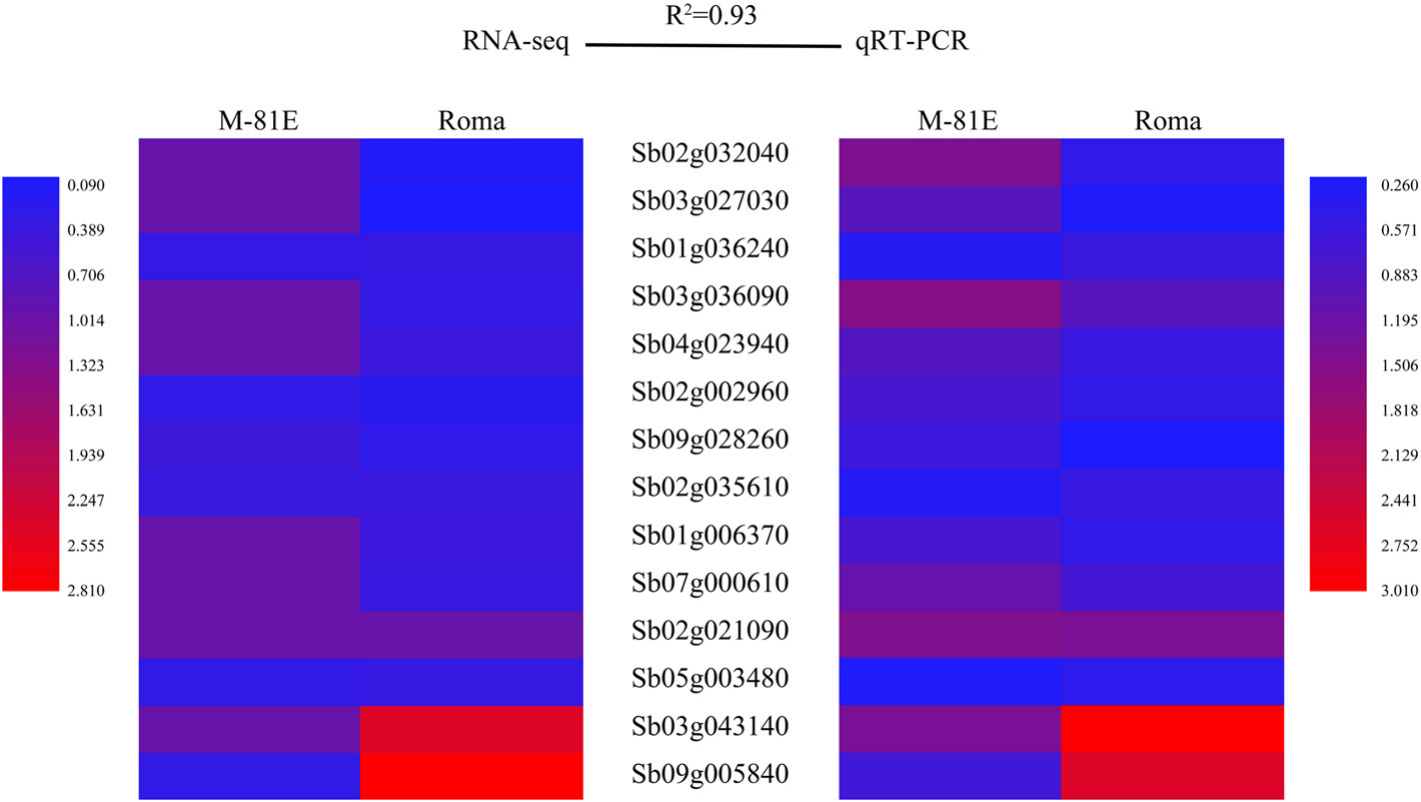
Validation of RNA-seq results by RT-qPCR. Expression levels of 14 randomly selected genes in the four samples used in this study were detected by RT-qPCR. R^2^ represents the correlation coefficient value between the two platforms. The numbers in the scale bar stand for RPKM values in RNA-seq and ΔΔCt in qRT-PCR, which were used to evaluate the correlation (R^2^). Primers are listed in (Additional file 9: Table S2)

**Fig. 11:**
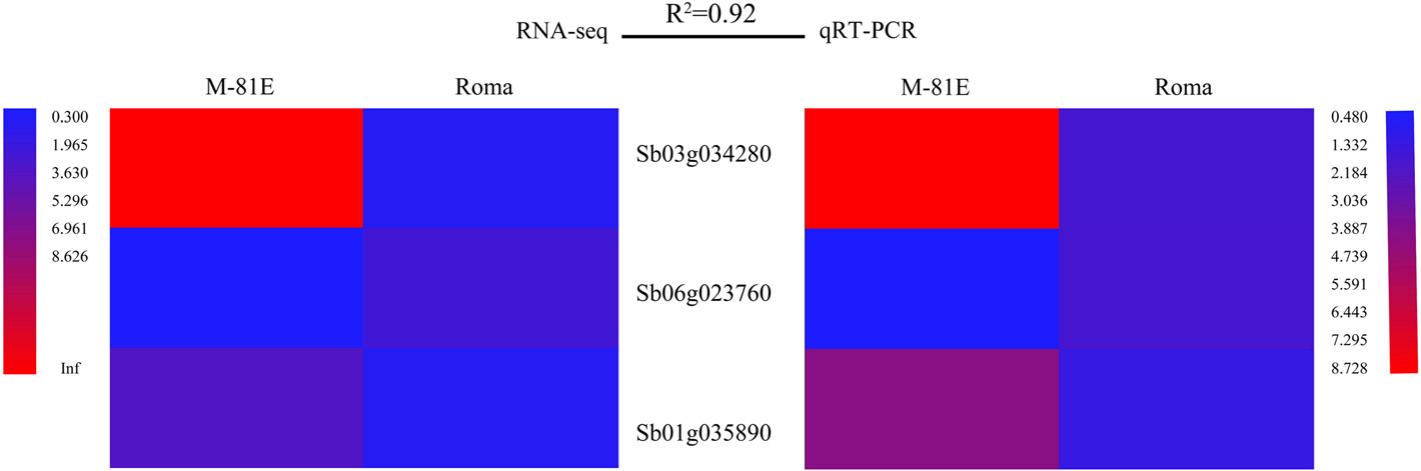
Validation of RNA-seq results by RT-qPCR. Expression levels of 3 genes involved in sucrose synthesis and metabolism pathways were detected by RT-qPCR. R^2^ represents the correlation coefficient value between the two platforms. The numbers in the scale bar stand for RPKM values in RNA-seq and ΔΔCt in qRT-PCR, which were used to evaluate the correlation (R^2^). Primers are listed in (Additional file 9: Table S2)

We performed quantitative real-time PCR on 14 randomly selected DEGs to validate the RNA-seq gene expression analysis. As shown in Fig. 10, a high correlation (R^2^ = 0.93) between RNA-seq and qRT-PCR was observed. Also, three genes (Sb03g034280, Sb06g023760 and Sb01g035890) which may play important roles in improving sugar content in sweet sorghum were confirmed by qPCR, too. As shown in Fig. 11, a high correlation (R^2^ = 0.92) was observed, confirming the reliability of the RNA-seq data.

## Discussion

Sweet sorghum has been considered to be a plant with relatively high salt tolerance [9, 12, 44]. In our work, however, NaCl caused a dramatic decrease in leaf length, leaf number, FW and DW in Roma, while growth parameters in M-81E were less affected (Fig. 1, Additional file 1: Figure S1 and Additional file 2: Figure S2). These results were consistent with previous studies that M-81E was observed to be relatively salt-tolerant but Roma was salt-sensitive.

Generally, the effects of salt stress include ion toxicity and osmotic stress. Ion toxicity is mainly caused by Na^+^. Increases in Na^+^ concentration during salt stress have been well established [45, 46]. Since sweet sorghum has the ability to exclude toxic ions and store the absorbed toxic ions in the root cell vacuoles while maintaining higher levels of K^+^ uptake [9], the accumulation of Na^+^ in leaves can be limited. In the present study, the accumulation of Na^+^ in leaves was not significantly affected in both our tested genotypes under 50 mM NaCl stress condition for 7 days. At 150 mM NaCl, however, Na^+^ concentration was significantly increased in both genotypes, particularly in Roma by a factor of six (Additional file 3: Figure S3). High concentration of Na^+^ impairs the ability of plants to accumulate essential nutrients [47], such as K^+^, which are required to maintain the stability and functioning of cell membranes and associated enzymes. Maintenance of adequate K^+^ levels in plant tissues under salt stress has been reported to be dependent on selective cellular K^+^ and Na^+^ distribution [48]. K^+^ content decreased with raised NaCl concentration and as a result, the K^+^/Na^+^ ratios decreased. These results indicated that sweet sorghum limited accumulation of Na^+^ under 50 mM NaCl treatment, while at a NaCl concentration increased of 150 mM, Roma lost the ability to control the absorption of Na^+^.

In order to compare the salt response of sugar content in leaves of M-81E and Roma at the transcriptome level, RNA-seq was performed using leaves treated with 0 and 150 mM NaCl for 48 h. In response to salt stress, a larger number of DEGs were observed in Roma compared to M-81E (Fig. 6). In a previous study, the results showed that the salt tolerant plants had a smaller number of salt-regulated genes in salt cress [49]. However, another study indicated that a salt-sensitive tomato PI365967 showed a relatively smaller amount of salt-regulated genes than Moneymaker which is more tolerant to salt [22]. These results suggested that the number of salt-regulated genes may not be directly linked to the degree of salt tolerance.

Pathway analysis is an effective way to characterize “gene networks” under salt stress. We observed that genes related with photosynthesis and light-harvesting proteins were mainly repressed by salinity. There were 20 DEGs mapped to the photosynthesis pathway in Roma and all of them were down-regulated under salt stress. Most of these genes were related to the structure of the photosystem complex, the electron transport chain and the connection between photosystem complexes and light-harvesting proteins (Table 1). Only 11 DEGs in M-81E mapped to the photosynthesis pathway with 9 down-regulated genes and 2 up-regulated genes, which were related to the stable assembly of the oxygen-evolving complex and ATP synthase. These results suggest that salt stress could damages the structure of the photosystem and reduces the efficiency of electron transportation, which may result in decreased ATP and NADPH levels in plants under salt stress. This damage is particularly severe in salt-sensitive species, while salt-tolerant species can protect important connective structures from being destroyed by keeping low concentration of Na^+^ in leaves and increasing the expression of particular genes. As a result, there was no significant change in Fv/Fm of M-81E, while Fv/Fm of Roma decreased gradually with the increasing NaCl treatments. Furthermore, the decrease in ΦPSII was more significant in Roma (Fig. 2). There were 7 and 13 DEGs mapped to the photosynthesis-antenna proteins pathway in M-81E and Roma, respectively. All of these genes were down-regulated under salt stress (Table 1), suggesting that the ability to capture and convert light energy of both genotypes was affected by salt stress. As light-harvesting complexes contain most of the chlorophyll and carotenoid pigments, the down-regulation of those genes resulted in decreases in chlorophyll content (Fig. 3), particularly in the salt-sensitive genotype Roma.

Rubisco, PEPC and PPDK are well known as the key enzymes in the dark reaction of photosynthesis. Rubisco plays an important role in CO_2_ assimilation. Salt stress led to a reduced expression of Rubisco encoding gene in both M-81E and Roma, suggesting that salt stress reduced the efficiency of CO_2_ assimilation in sweet sorghum as has been shown in previous studies [50–53]. To our surprise, the expression of the gene encoding NADP-ME was extremely enhanced by salt stress in M-81E. NADP-ME is important for the carbon fixation pathway because it catalyzes the reversible oxidative decarboxylation of L-malate to produce CO_2_, pyruvate and NADPH [54, 55]. It has been shown that the expression of the gene encoding NADP-ME can be activated by salt stress [56, 57]. NADP-MEs are not only an important for photosynthesis, but are also involved in plant defense reactions and environmental stress responses [58]. In our study, the gene encoding NADP-ME was up-regulated by salt stress in M-81E which could increase the content of CO_2_, pyruvate and NADPH. The increasing CO_2_ and pyruvate levels enhanced the efficiency of the dark reaction of photosynthesis. In our research, photosynthetic rate of M-81E was not significantly affected by salt stress (Fig. 4), which might be related to the up-regulation of *NADP-ME*. After treated with 150 mM NaCl, in Roma, photosynthetic rate and stomatal conductance decreased, while the intracellular CO_2_ concentration increased (Fig. 4). This showed that the decrease of photosynthetic rate was attributed to the non-stomatal factors, which might be related to the down expression of Rubisco. Recent studies showed that *NADP-ME* plays a role in enhancing tolerance of plants to salt stress [57, 59]. Salt stress can produce superabundant reactive oxygen species (ROS) causing oxidative stress in plants [60–62]. Additionally, NADPH provides the reducing power required for ROS metabolism [63]. Møller and Rasmusson (1998) reported that NADPH can be used by the NADPH-specific glutathione reductase (GR) to catalyze the reduction of glutathione for scavenging ROS by an ascorbate coupled system [64]. In our study, the up-regulation of NADP-ME encoding gene may play a role in the stress response and in the dark reaction of photosynthesis in salt-tolerant species of sweet sorghum. The increase of NADP-ME content enhanced the recycling of CO_2_ in the C4 pathway. Furthermore, it may reduce the damage caused by ROS.

After treated with NaCl for 7 days, there was a significant difference in sugar content between M-81E and Roma. The sugar content increased 99.7 % in M-81E and decreased 30.5 % in Roma under 150 mM NaCl, suggesting that salt stress strongly induced the accumulation of sugar in salt-tolerant genotype of sweet sorghum (Fig. 5). Sucrose is the main source of carbon and of energy the sink tissues of sweet sorghum [8]. The cytoplasm of leaves is the site for sucrose synthesis. After synthesized, sucrose will be loaded into phloem and transported to sink tissues (stem and/or panicle). Various enzymes involved in sugar metabolism are required to ensure that sucrose is synthesized efficiently and the flow of sucrose is unidirectional (from source to the sink) [65]. SS is known to play a role in sucrose synthesis and its activity is high in source tissues such as leaves [43]. SPS can synthesize sucrose phosphate, which is converted to sucrose by sucrose phosphate phosphatase (SPP) in source tissues and then loaded into phloem. INV plays the most important role in the decomposition of sucrose. The vacuolar invertase activity is high in rapidly growing tissues [66]. Sucrose transporters (SUTs) are important exporters of photosynthetically-produced sugar, principally sucrose, from leaves to sink tissues [67]. It has been reported that the expression of sucrose-metabolizing enzymes play an important role in the accumulation of sucrose. In our study, the SS gene was up-regulated only in M-81E by salt treatment. However, the gene encoding INV was found to be up-regulated in Roma but down-regulated in M-81E (Additional file 8: Figure S7, Table 1). These findings suggest that salt-tolerant species of sweet sorghum accumulate more sucrose by enhancing the synthesis and reducing the decomposition of sucrose under salt stress. While, salt-sensitive species enhance decomposition of sucrose under salt stress in order to meet the energy demand of growth. Furthermore, genes encoding SUTs showed no differential expression after treated with 150 mM NaCl in either genotype, suggesting that the transportation of sucrose from leaves to stem is not affected by salt stress treatment for 48 h at seedling stages.

## Conclusions

**Fig. 12:**
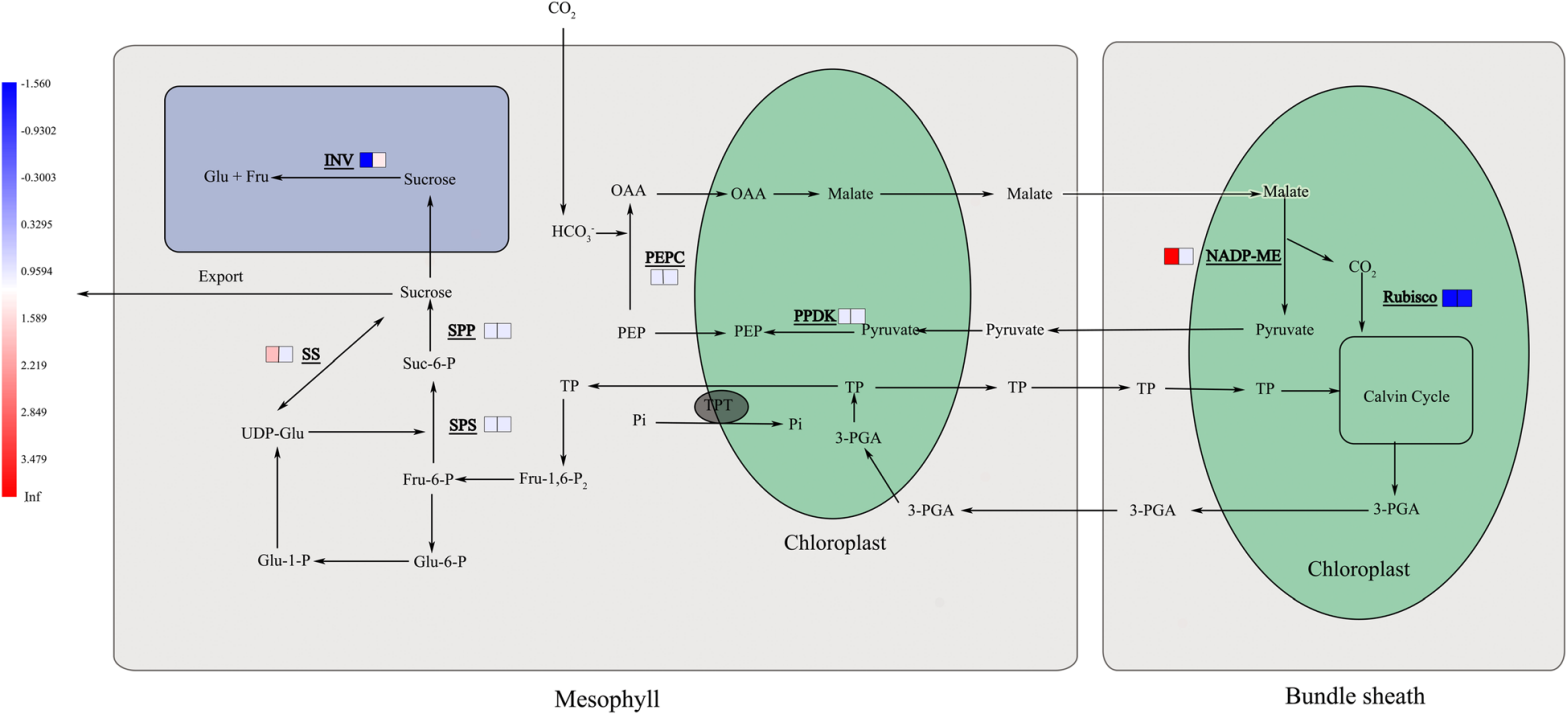
visualization of DEGs involved in pathways related with the accumulation of sugar. A square block represents a gene assigned to our RNA-seq data. Blue represents the gene was down-regulated in salt-treated samples compared to the control samples. Red represents the gene was up-regulated. For each gene, the square block on the left stand for M-81E and the right one stand for Roma

In conclusion, we report here that the salt-tolerant genotype M-81E can increase sugar content under salt stress. This may be caused by the changes in expression level of genes related to important structures of photosystems and LHCs and genes encoding key enzymes of sucrose synthetase and sucrose decomposition under salt stress (Fig. 12). This RNA-seq dataset is an important resource for future studies aimed at improving sugar content of sweet sorghum under salt stress. Further genetic and biochemical analysis will be critical to understanding the detailed gene function and the relationship between salt tolerance and sugar content in sweet sorghum.

## Methods

### Plant materials and growth conditions

Seeds of two sweet sorghum genotypes M-81E and Roma were used as the experimental materials in this study. M-81E is considered to be tolerant to salt stress [9], while Roma is sensitive to salt stress. After being washed with tap water for 8 h, plump seeds were selected and sowed in plastic pots filled with river sand and irrigated with tap water. After germination, they were irrigated with 1/2 Hoagland solution in controlled growth chambers. The seedlings were cultured at 28 ± 3/23 ± 3 °C (day/night) at a light intensity of 600 μmol m^−2^ s^−1^ (15 h photoperiod) and 70 % relative humidity. Salt treatment was performed at the four-leaf stage. The treated plants were irrigated with nutrient solution supplemented with 0, 50 and 150 mM NaCl. The NaCl concentrations were increased stepwise towards the final concentrations by 50 mM each day.

### Measurement of fresh weight and dry weight

After exposure to salt treatments for 7 days, 15 plants from each treatment (5 per replicate) were sampled to determine leaf length and leaf number. Leaves were then separated and their FW were directly determined. For DW determination, the leaves were weighed after being dried at 150 °C for 15 min and 70 °C for 72 h. Water content (WC) was defined as follows: WC (%) = [(FW − DW)/FW × 100.

### Analysis of inorganic ions

For Na^+^ and K^+^ analysis, samples of dried leaves were ashed in a furnace for 6 h at 500 °C. The ash was dissolved in 20 % nitric acid, diluted in distilled water and filtered through a sheet of filter paper. Na^+^ and K^+^ contents were determined by flame emission photometry (Flame Photometer 410,UK). Inorganic ion contents were expressed in mg g^−1^ DW.

### Measurements of chlorophyll fluorescence

After exposure to salt treatments for 7 days, chlorophyll fluorescence was measured. For each treatment, the parameters of Chl fluorescence were measured independently on five plants. Measurements were taken on the mature leaves of each of the chosen plants. Chl fluorescence was measured using a portable fluorometer (FMS2, Hansatech, King’s Lynn, UK) following the protocol described by Kooten and Snel [68]. Leaves had been pre-darkened for at least 1 h in order to determine the minimal and the maximal fluorescence. Minimal fluorescence (Fo) with all PSII reaction centers open was determined by modulated light which was low enough not to induce any significant variable fluorescence (Fv). Maximal fluorescence (Fm) [43] with all reaction centers closed was determined by 0.8 s saturating light of 8000 μmol m^−2^ s^−1^ on a dark-adapted leaf. Then the leaf was illuminated by an actinic light of 500 μmol m^−2^ s^−1^. Steady-state fluorescence (Fs) was recorded when the leaf reached steady-state photosynthesis. A second 0.8 s saturating light of 8000 μmol m^−2^ s^−1^ was given to determine maximal fluorescence in the light-adapted state (Fm’). Maximal photochemical efficiency (Fv/Fm) of PSII was expressed as: Fv/Fm = (Fm–Fo)/Fm. Quantum yield of PSII electron transport was: ΦPSII = (Fm’–Fs)/Fm’.

### Measurement of chlorophyll content

Leaves (0.2 g FW) were washed in distilled water and extracted in 5 ml 80 % acetone and 5 ml dimethyl sulfoxide at 65 °C in darkness for 24 h. The extract was adjusted to a total volume of 25 ml with 80 % acetone. The absorbance of the extract was determined at 663 and 645 nm using a spectrophotometer of BECKMAN DU2600. The amount of total chlorophyll was calculated using the Arnon [69] formulae as follows: Chla(mg/g) = (12.7A_663_-2.69A_645_) × V/1000 W; Chlb(mg/g) = (22.9A_645_-4.68A_663_) × V/1000 W; Chl(mg/g) = (8.02A_663_ + 20.21A_645_) × V/1000 W. “V” represented the volume of the extract solution of 25 ml, “W” represented the weight of the sampling leaves of 0.2 g.

### Measurement of photosynthesis

photosynthetic rate, stomatal conductance and intercellular CO_2_ concentration were measured using Li-6400 photosynthesis measurement system. Measurements were taken on the mature leaves of each of the chosen plants.

### Measurement of sugar content

After exposure to salt treatments for 7 days, 15 plants from each treatment (5 per replicate) were sampled to determine sugar content. Sugar content were determined by the anthrone method described by Spiro [70]. 100 μl leaf extract were added to 3 ml (final volume) assay media containing 1.08 M H_2_SO_4_, 1.09 mM thiourea and 2.1 mM anthrone. The mixture was heated at 100 °C for 10 min and absorbance read at 620 nm. A calibration curve with D-glucose was done as a standard.

### Total RNA extraction

Total RNA was isolated from the leaves of sweet sorghum of each genotype treated with 0 and 150 mM NaCl for 48 h using a Total Plant RNA Extraction Kit (Karroten, Beijing, China) following the manufacturer's protocols. The RNA was quantified using a Nanodrop ND-1000 spectrophotometer (Thermo Fisher Scientific, Wilmington, DE, USA). A 1 % agarose gel buffered by Tris–acetate–EDTA was also run to determine the integrity of the RNA.

### Library construction and Illumina sequencing

Libraries were constructed following a High Throughput Illumina Strand-Specific RNA Sequencing Library protocol [71]. Briefly, mRNA was purified from 5 μg of total RNA using oligo (dT) magnetic beads. The purified mRNA was fragmented into small pieces using fragmentation buffer. Taking these short fragments as templates, first-strand cDNA was synthesized using reverse transcriptase and random hexamer primers. Second-strand cDNA synthesis was followed using DNA polymerase I and RNase H. Sequencing adapters were ligated to short fragments after purification with the QiaQuick PCR extraction kit, and which were used to distinguish different sequencing samples. Fragments with different lengths were then separated by agarose gel electrophoresis and selected for PCR amplification as sequencing templates. The final cDNA library was sequenced using Illumina HiSeq™ 2500 at **BioMarker Technologies Co** Ltd, Beijing. RNA-seq data of the untreated control and salt-treated samples were obtained from two and three biological replicates, respectively. The raw reads were cleaned by removing adaptor sequences, empty reads and low quality sequences. Then, clean reads were generated.

### Mapping and detection of DEGs

Clean reads were mapped to the sorghum genome [72, 73] using TopHat version 2.0.10 [74]. Mapping results generated by TopHat were filtered to retain only unique mapped reads before being piped into Cuffdiff (http://cole-trapnell-lab.github.io/cufflinks/) to estimate read counts for each gene. Reads per KB per million (RPKM) values were calculated by an in-house script based on the count table of Cuffdiffs output. The RPKM measure of read density reflects the molar concentration of a transcript in the starting sample by normalizing for RNA length and for the total read number in the measurement. A RPKM threshold value of 0.1was set to detect the presence of a transcript for a particular gene. DEGs were defined using DESeq [75] as fold changes≧2 with a false discovery rate (FDR) adjusted p value ≤0.01.

### Gene annotation and classifications

The optimal assembly results were chosen according to the assembly evaluation. The assembled sequences were compared against the NCBI non-redundant (nr) database [76], Swiss-Prot [77], GO [23], COG [24] and KEGG [25] database using BLAST [78] with E-value ≦ 1e-10 as the cutoff. To annotate the assembled sequences with GO terms, the Swiss-Prot BLAST results were imported into Blast2GO [79]. These GO terms were assigned to query sequences, producing a broad overview of groups of genes catalogued in the transcriptome for each of three ontology vocabularies, biological processes, molecular functions [50] and cellular components [23]. The unigenes sequences were also aligned to the COG database to predict and classify functions. KEGG pathways were assigned to the assembled sequences using the online KEGG web server (http://www.genome.jp/kegg/). The output of KEGG analysis includes KO assignments and KEGG pathways that are populated with the KO assignments.

### Quantitative real-time PCR analysis

Fourteen DEGs were randomly selected for quantitative real-time PCR to verify the RNA-seq results. Also, three genes which may play important roles in improving sugar content in sweet sorghum were confirmed by qPCR, too. Primers for these 17 genes were designed using the Beacon Designer software (version 7.0) (Additional file 9: Table S2). *S. bicolor*’s housekeeping gene β-actin (GenBank ID: X79378) was used as an internal standard. 1 μg of total RNA was used per 20 μl reaction for reverse transcription. Polymerase chain reaction was performed in a 20 μl reaction mixture with 10 μl SYBR Premix Ex Taq (Bio-RAD, California, USA), 0.5 μl of both forward and reverse primers, 7 μl of double distilled H_2_O and 2 μl (40 ng/μl) of the cDNA. Real-time PCR was performed on a real-time quantitative PCR instrument (Bio-RAD, California, USA). 2^-△△Ct^ method was used to calculate the relative expression of each gene [80].

### Statistical analysis

Multiple comparisons were performed between different samples using Duncan’s test at the 0.05 significance level. All tests were performed with SPSS Version 16.0 for Windows (SPSS, Chicago. IL, USA).

### Availability of supporting data

The data sets supporting the results of this article are included within the article and its additional files. The reads produced in this study have been deposited in the National Center for Biotechnology Information (NCBI) SRA database with accession number of SRX1048181, SRX1050048 for M-81E control, SRX1050049, SRX1050050 for M-81E salt-treated, SRX1050054, SRX1050055 for Roma control, and SRX1050056, SRX1050057 for Roma salt-treated. Access to the data is available upon publication at http://www.ncbi.nlm.nih.gov/sra/.

## Additional files

Additional file 1: Figure S1. Effect of increasing NaCl concentration on leaf length and leaf number of Roma and M-81E under 3 salt treatments (0, 50 and 150 mM) for 7 days. Values are means ± SD of five replicates. Bars with the different letters are significantly different at *p* = 0.05 according to Duncan’s multiple range test. Additional file 2: Figure S2. Fresh weight, dry weight and water content of M-81E and Roma treated with different concentrations of NaCl (0, 50 and 150 mM) for 7 days. Values are means ± SD of five replicates. Bars with the different letters are significantly different at *p* = 0.05 according to Duncan’s multiple range test. Bars with same letter are not significantly different. Additional file 3: Figure S3. Concentration of Na^+^, K^+^ and the K^+^/Na^+^ ratio in leaves of Roma and M-81E under 3 salt treatments (0, 50 and 150 mM) for 7 days. Values are means ± SD of five replicates. Bars with the different letters are significantly different at *p* = 0.05 according to Duncan’s multiple range test. Bars with same letter are not significantly different. Additional file 4: Table S1. Clean reads used for further analysis. Additional file 5: Figure S4. Clusters of orthologous groups (COG) classification. Additional file 6: Figure S5. KEGG map of the photosynthesis- antenna proteins pathway. It’s an analysis of DEGs, comparing salt-treated samples to untreated control. Boxes with a red frame indicate the corresponding DEGs were up-regulated in the salt-treated samples, boxes with a green frame indicate the corresponding DEGs were down-regulated in the salt-treated samples, boxes with blue frame indicate some of the corresponding DEGs were down-regulated and others were up-regulated, and those without any colored frame indicate the expression level of corresponding genes were not changed, as determined by RNA-seq. Additional file 7: Figure S6. KEGG pathway analysis of the carbon fixation in photosynthetic organisms pathway of M-81E (A) and Roma (B). It’s an analysis of DEGs, comparing salt-treated samples to untreated control. The number in each box represents enzyme commission number. Boxes with a red frame indicate the corresponding DEGs were up-regulated in the salt-treated samples, boxes with a green frame indicate the corresponding DEGs were down-regulated in the salt-treated samples, boxes with blue frame indicate some of the corresponding DEGs were down-regulated and others were up-regulated, and those without any colored frame indicate the expression level of corresponding genes were not changed, as determined by RNA-seq. Additional file 8: Figure S7. KEGG pathway analysis of the starch and sucrose metabolism pathway of M-81E (A) and Roma (B). It’s an analysis of DEGs, comparing salt-treated samples to untreated control. The number in each box represents enzyme commission number. Boxes with a red frame indicate the corresponding DEGs were up-regulated in the salt-treated samples, boxes with a green frame indicate the corresponding DEGs were down-regulated in the salt-treated samples, boxes with blue frame indicate some of the corresponding DEGs were down-regulated and others were up-regulated, and those without any colored frame indicate the expression level of corresponding genes were not changed, as determined by RNA-seq. Additional file 9: Table S2. Table S2 Primer pairs for real-time quantitative PCR.

## Abbreviations

OAA: Oxaloacetate
TP: Triose phosphate
TPT: Triose phosphate translocator
SPS: Sucrose phosphate phosphatase
SS: Synthase
INV: Invertase
SUT: Sucrose transporters
FW: Fresh weights
DW: Dry weight
WC: Water content
NADP-ME: NADP^+^-malate enzyme
RNA-seq: RNA-sequencing
DEGs: Differentially expressed genes
RPKM: Reads per KB per million
FDR: False discovery rate
GO: Gene ontology
COG: Clusters of orthologous groups
KEGG: Kyoto encyclopedia of genes and genomes
LHCs: Light-harvesting complexes
rubisco: Ribulose-bisphosphate carboxylase
PEPC: Phosphoenolpyruvate carboxylase
PPDK: Pyruvate orthophosphate dikinase
