# Supplementary figures and images for: Identification and transcriptomic profiling of genes involved in increasing sugar content during salt stress in sweet sorghum leaves
Source: BMC Genomics. 2015 Jul 19;16(1):534. doi: 10.1186/s12864-015-1760-5 (PMC4506618; doi:10.1186/s12864-015-1760-5)

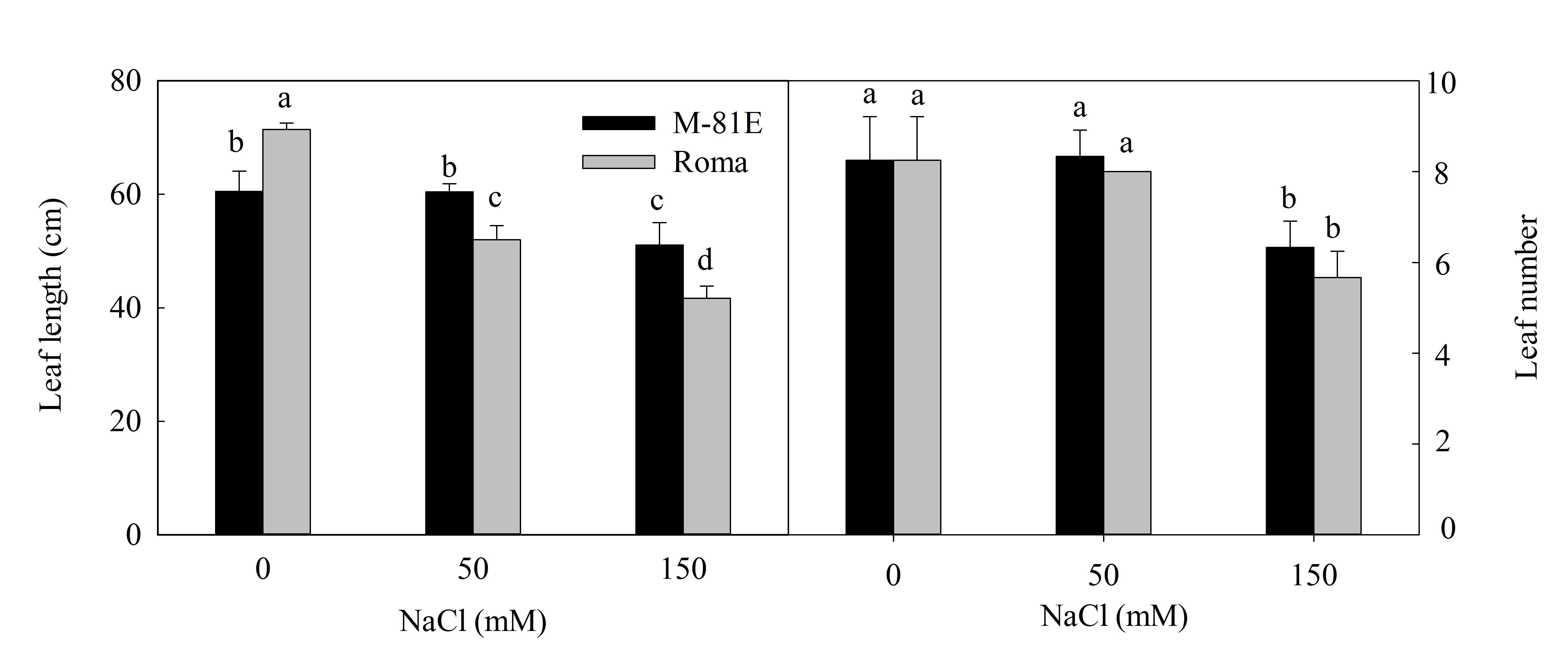

Supplement: Additional file 1: Figure S1. — Effect of increasing NaCl concentration on leaf length and leaf number of Roma and M-81E under 3 salt treatments (0, 50 and 150 mM) for 7 days. Values are means ± SD of five replicates. Bars with the different letters are significantly different at p = 0.05 according to Duncan’s multiple range test. [file 12864_2015_1760_MOESM1_ESM.jpg]

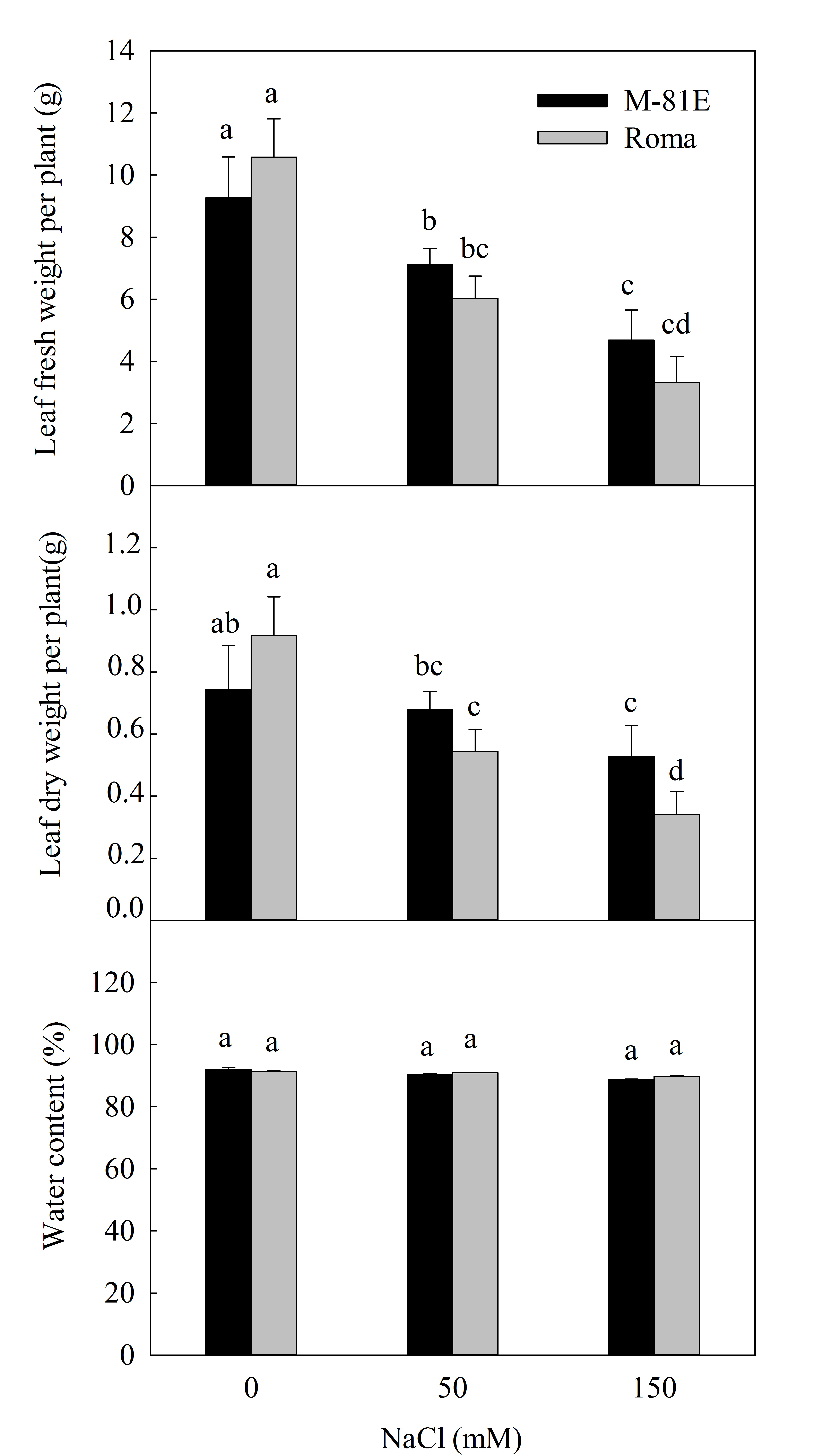

Supplement: Additional file 2: Figure S2. — Fresh weight, dry weight and water content of M-81E and Roma treated with different concentrations of NaCl (0, 50 and 150 mM) for 7 days. Values are means ± SD of five replicates. Bars with the different letters are significantly different at p = 0.05 according to Duncan’s multiple range test. Bars with same letter are not significantly different. [file 12864_2015_1760_MOESM2_ESM.jpg]

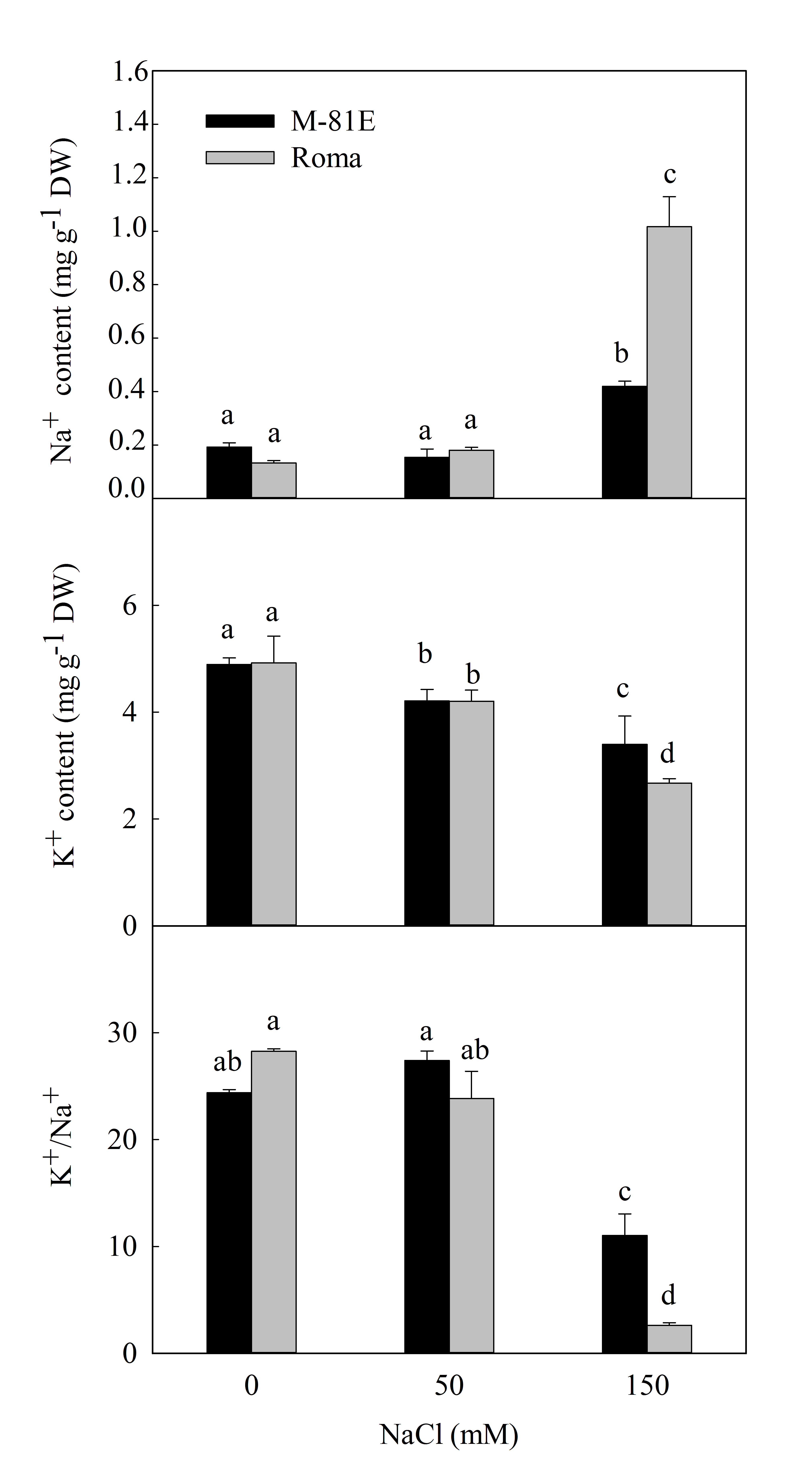

Supplement: Additional file 3: Figure S3. — Concentration of Na+, K+ and the K+/Na+ ratio in leaves of Roma and M-81E under 3 salt treatments (0, 50 and 150 mM) for 7 days. Values are means ± SD of five replicates. Bars with the different letters are significantly different at p = 0.05 according to Duncan’s multiple range test. Bars with same letter are not significantly different. [file 12864_2015_1760_MOESM3_ESM.jpg]

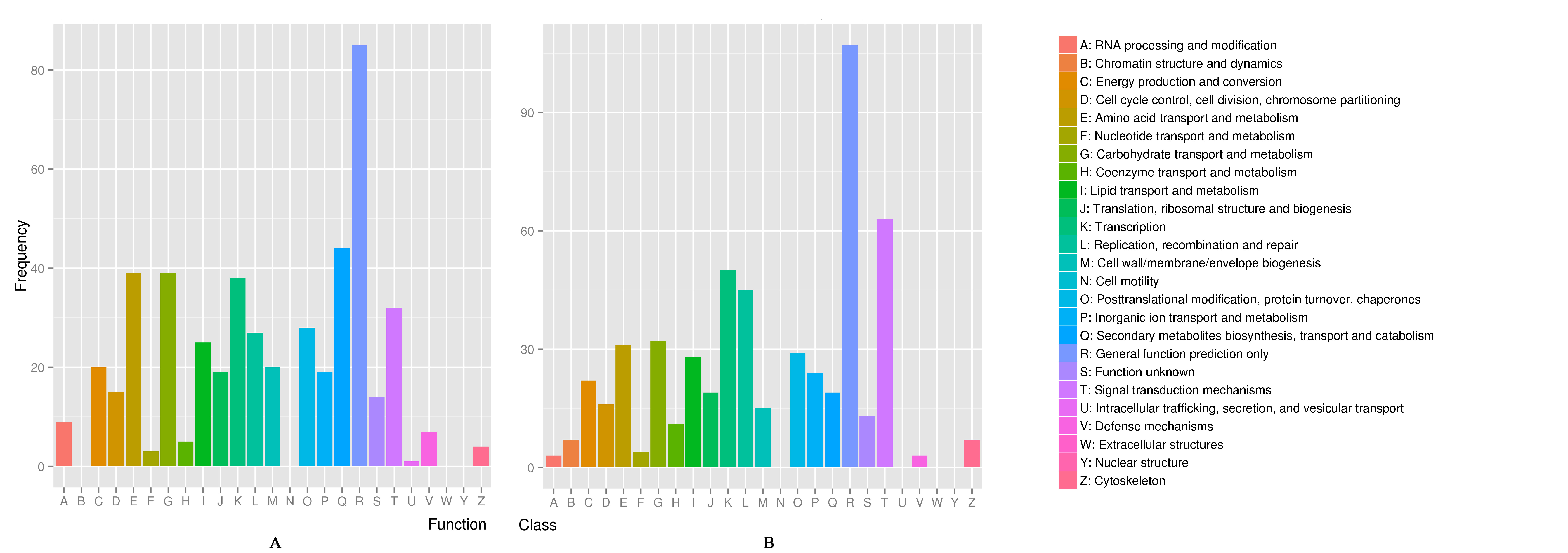

Supplement: Additional file 5: Figure S4. — Clusters of orthologous groups (COG) classification. [file 12864_2015_1760_MOESM5_ESM.jpg]

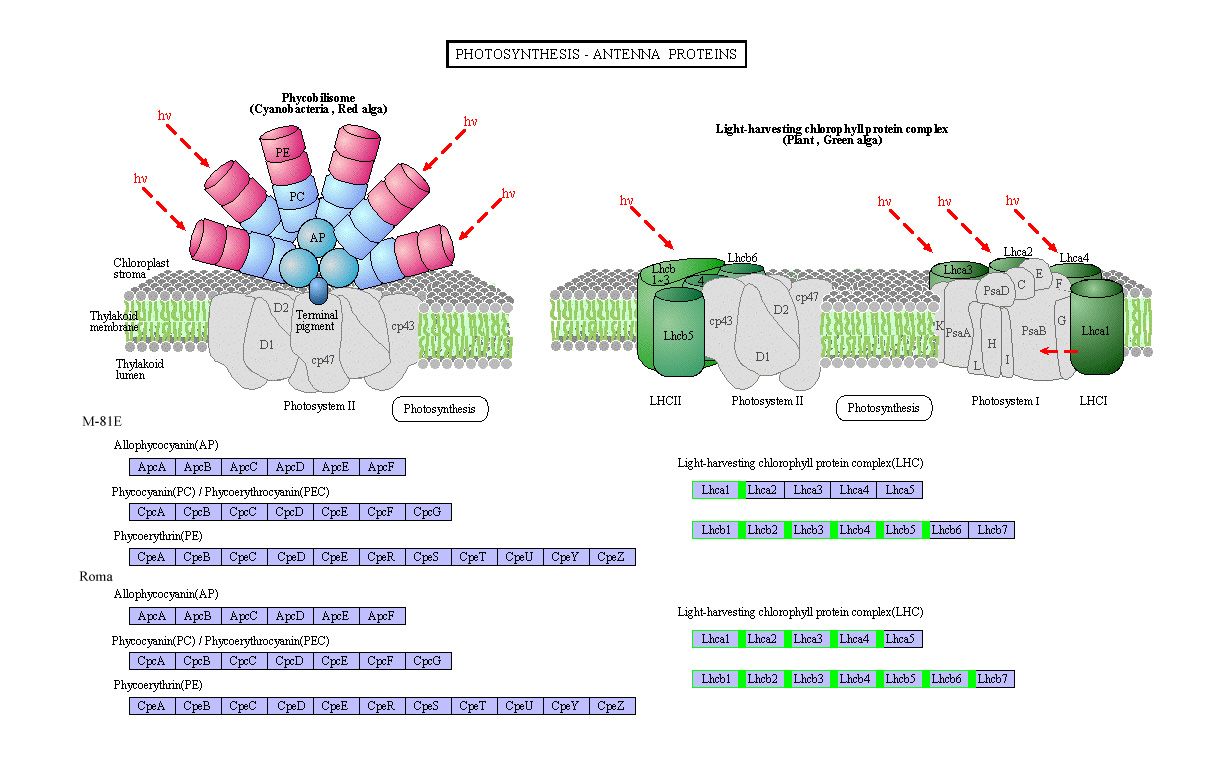

Supplement: Additional file 6: Figure S5. — KEGG map of the photosynthesis- antenna proteins pathway. It’s an analysis of DEGs, comparing salt-treated samples to untreated control. Boxes with a red frame indicate the corresponding DEGs were up-regulated in the salt-treated samples, boxes with a green frame indicate the corresponding DEGs were down-regulated in the salt-treated samples, boxes with blue frame indicate some of the corresponding DEGs were down-regulated and others were up-regulated, and those without any colored frame indicate the expression level of corresponding genes were not changed, as determined by RNA-seq. [file 12864_2015_1760_MOESM6_ESM.jpg]

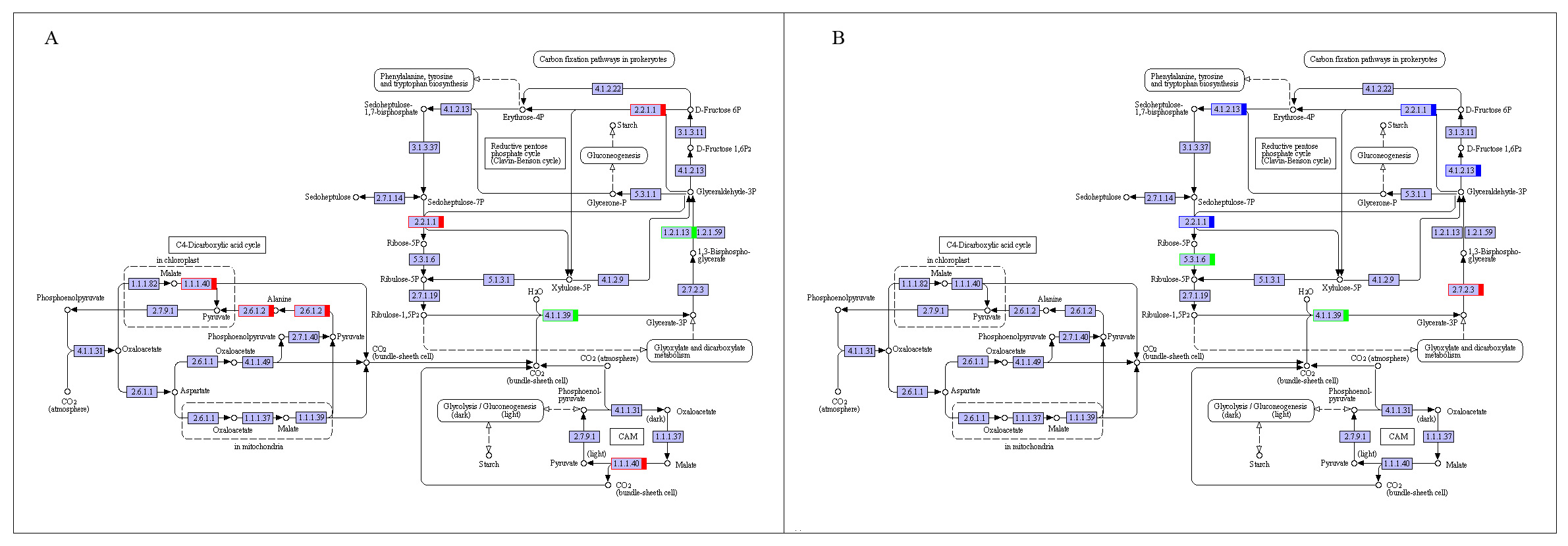

Supplement: Additional file 7: Figure S6. — KEGG pathway analysis of the carbon fixation in photosynthetic organisms pathway of M-81E (A) and Roma (B). It’s an analysis of DEGs, comparing salt-treated samples to untreated control. The number in each box represents enzyme commission number. Boxes with a red frame indicate the corresponding DEGs were up-regulated in the salt-treated samples, boxes with a green frame indicate the corresponding DEGs were down-regulated in the salt-treated samples, boxes with blue frame indicate some of the corresponding DEGs were down-regulated and others were up-regulated, and those without any colored frame indicate the expression level of corresponding genes were not changed, as determined by RNA-seq. [file 12864_2015_1760_MOESM7_ESM.jpg]

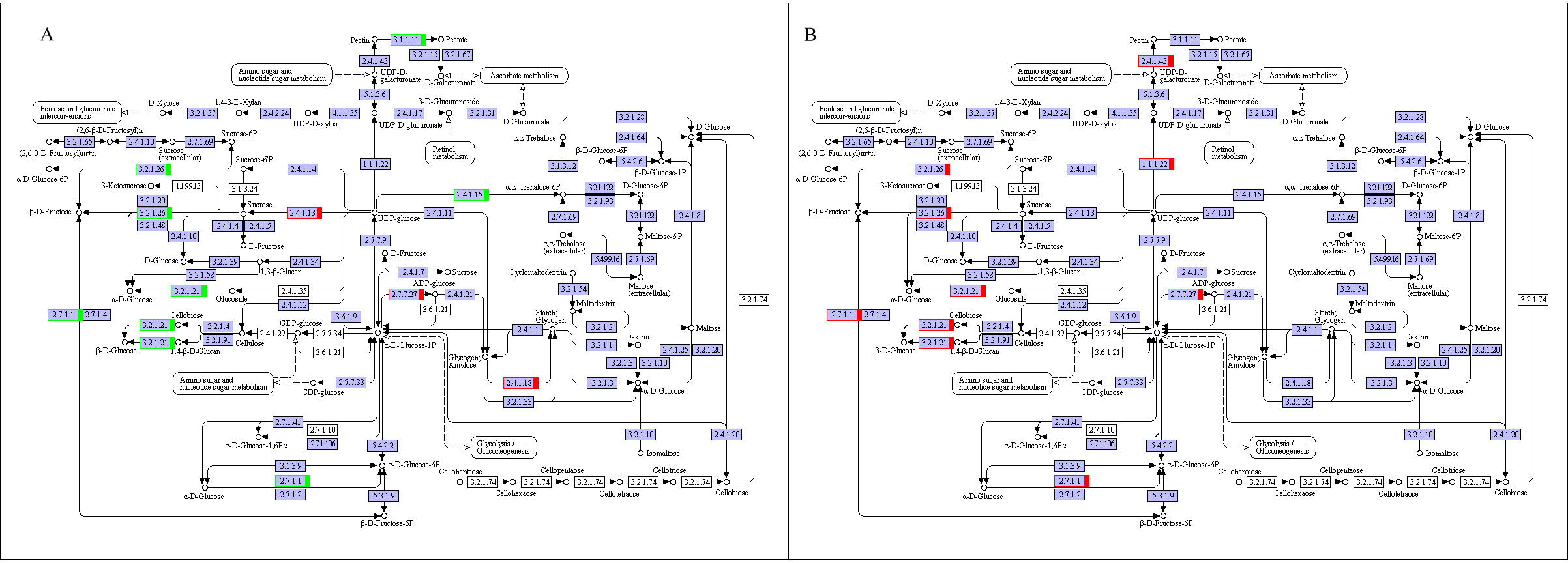

Supplement: Additional file 8: Figure S7. — KEGG pathway analysis of the starch and sucrose metabolism pathway of M-81E (A) and Roma (B). It’s an analysis of DEGs, comparing salt-treated samples to untreated control. The number in each box represents enzyme commission number. Boxes with a red frame indicate the corresponding DEGs were up-regulated in the salt-treated samples, boxes with a green frame indicate the corresponding DEGs were down-regulated in the salt-treated samples, boxes with blue frame indicate some of the corresponding DEGs were down-regulated and others were up-regulated, and those without any colored frame indicate the expression level of corresponding genes were not changed, as determined by RNA-seq. [file 12864_2015_1760_MOESM8_ESM.jpg]
